# An Immunity-Related Gene Model Predicts Prognosis in Cholangiocarcinoma

**DOI:** 10.3389/fonc.2022.791867

**Published:** 2022-07-01

**Authors:** Han Guo, Yihan Qian, Yeping Yu, Yuting Bi, Junzhe Jiao, Haocheng Jiang, Chang Yu, Hailong Wu, Yanjun Shi, Xiaoni Kong

**Affiliations:** ^1^ Department of Oncology, Renji Hospital, School of Medicine, Shanghai Jiao Tong University, Shanghai, China; ^2^ Institute of Clinical Immunology, Department of Liver Diseases, Central Laboratory, Shuguang Hospital Affiliated to Shanghai University of Chinese Traditional Medicine, Shanghai, China; ^3^ Department of Liver Surgery, Renji Hospital, School of Medicine, Shanghai Jiao Tong University, Shanghai, China; ^4^ Shanghai Key Laboratory for Molecular Imaging, Collaborative Research Center, Shanghai University of Medicine and Health Sciences, Shanghai, China; ^5^ Department of Hepatobiliary and Pancreas Surgery , The Second Affiliated Hospital of Zhejiang University School of Medicine, Hangzhou, China

**Keywords:** immunity, prognosis, cholangiocarcinoma, TCI, LASSO

## Abstract

The prognosis of patients with cholangiocarcinoma (CCA) is closely related to both immune cell infiltration and mRNA expression. Therefore, we aimed at conducting multi-immune-related gene analyses to improve the prediction of CCA recurrence. Immune-related genes were selected from the Gene Expression Omnibus (GEO), The Cancer Genome Atlas (TCGA), and the Immunology Database and Analysis Portal (ImmPort). The least absolute shrinkage and selection operator (LASSO) regression model was used to establish the multi-gene model that was significantly correlated with the recurrence-free survival (RFS) in two test series. Furthermore, compared with single genes, clinical characteristics, tumor immune dysfunction and exclusion (TIDE), and tumor inflammation signature (TIS), the 8-immune-related differentially expressed genes (8-IRDEGs) signature had a better prediction value. Moreover, the high-risk subgroup had a lower density of B-cell, plasma, B-cell naïve, CD8+ T-cell, CD8+ T-cell naïve, and CD8+ T-cell memory infiltration, as well as more severe immunosuppression and higher mutation counts. In conclusion, the 8-IRDEGs signature was a promising biomarker for distinguishing the prognosis and the molecular and immune features of CCA, and could be beneficial to the individualized immunotherapy for CCA patients.

## Introduction

Cholangiocarcinoma (CCA) is the second most common primary hepatic malignancy after hepatocellular carcinoma (HCC) (1), and the 5-year survival (7%–20%) and tumor recurrence rates after surgery are still disappointing in advanced CCA patients (2–4). In the last decade, targeted therapy and immunotherapy were applied to improve the clinical prognosis of patients. For example, the new targeted fibroblast growth factor receptor (FGFR) 2 inhibitor pemigatinib has been applied to the treatment of CCA; however, several factors including the emergence of polyclonal mutations determine resistance to pemigatinib and the identification of biomarkers predictive of response remain to be unexplored (5).

Immunotherapy is another therapeutic hotspot. The interplay between tumors and host immunity plays an important role in the progression of CCA. The high density of tumor-associated macrophages as the immunosuppressive element is linked to the increased tumor recurrence rate of CCA (6, 7), while the presence of CD4+ and CD8+ T cells has a significant relationship with favorable prognosis in CCA (8, 9). Therefore, cancer immunoediting has developed as a relevant hallmark and promotes tumor progression, which consists of three phases termed elimination, equilibrium, and escape. Throughout these phases, tumor immunogenicity is edited, and immunosuppressive mechanisms that promote cancer development are acquired (10, 11). Above all, immune checkpoint inhibitors (ICIs) are currently under investigation in advanced CCA; however, single-agent ICIs have reported controversial results in CCA, suggesting modest but real responses in a limited subset of patients (12).

To break through the bottleneck of immunotherapy in the subset of patients, many available preclinical CCA prediction models are applied to immunotherapy (13). However, each model has its limitations, and CCA progression is subject to the activity of the immune system, which varies among individuals. CCA transcriptome sequencing has verified that the subset of patients with an elevated tumor mutational load and upregulated immune checkpoint molecules has the poorest outcome (14). Therefore, features based on immune genes in CCA may be exploited for prognostic benefit.

Genome-wide profiling can be used for gaining insights into tumor progression, which is an efficient way to enhance our understanding of cancer biology (15–17). In this study, we sought to develop a CCA prognostic marker based on immune-related genes to improve the prognosis of CCA and provide reliable information for guiding the individual immunotherapy. Immune-related genes were selected from the Gene Expression Omnibus (GEO) (18), The Cancer Genome Atlas (TCGA) (19), and the Immunology Database and Analysis Portal (ImmPort) (20). The least absolute shrinkage and selection operator (LASSO) regression (21, 22) was used to establish the multi-gene model for predicting the recurrence-free survival (RFS) of CCA patients. To examine the prognostic ability of the model, it was compared with single genes, clinical characteristics, tumor immune dysfunction and exclusion (TIDE), and tumor inflammation signature (TIS). Furthermore, the multi-immune-related gene model was further verified in our own CCA specimens. These results might provide an efficient method for predicting recurrence, which might benefit the individualized immunotherapy of CCA patients

## Materials and Methods

### Preparation of CCA Datasets and Clinical CCA Specimens

GSE76297, GSE26566, GSE119336, and GSE89749 were downloaded from GEO. All of them met the criteria of having more than 20 samples, including both tumor and non-tumor samples, and the annotated genes accounted for more than 90% of the total transcriptomes (n > 17,000). The detailed information of these four datasets was listed in **Table 1**, and differentially expressed genes (DEGs) in the four databases were analyzed by the online analysis tool GEO2R (23). In addition, the clinical information of 36 CCA patients came from the TCGA database. Here, genes with adjusted *p*-value < 0.05 and |log2(fold change)| > 0.585 were considered as DEGs. The immune-related genes were collected from a database termed ImmPort. After cross-analysis of DEGs and immune genes, 151 dysregulated immune mRNAs were used for further research.

**Table 1:** GEO datasets enrolled in the study.

Database	Source	Sample			Platform
		**T**	**P**	**N**	
**GSE26566**	https://www.ncbi.nlm.nih.gov/geo/query/acc.cgi?acc=GSE26566	106	59	6	Illumina v2.0
**GSE119336**	https://www.ncbi.nlm.nih.gov/geo/query/acc.cgi?acc=GSE119336	15	–	15	Affymetrix 6.0
**GSE76297**	https://www.ncbi.nlm.nih.gov/geo/query/acc.cgi?acc=GSE76297	91	92	–	Affymetrix HTA-2_0
**GSE89749**	https://www.ncbi.nlm.nih.gov/geo/query/acc.cgi?acc=GSE89749	118	–	2	Illumina V4.0

N，normal ICC tissue samples; P, para-cancerous tissue samples; T, tumor samples.

From January 1, 2012, to December 30, 2018, the human CCA specimens were collected from the Department of Liver Surgery, Renji Hospital, Shanghai Jiaotong University. Patients who met the following criteria were included in the research: no preoperative radiotherapy, chemotherapy, and conservative treatment before surgery. Finally, the tissues of 45 patients were obtained, and all of them were pathologically confirmed. Protocols were approved and written informed consent was waived by the ethics review committee of Renji Hospital, School of Medicine, Shanghai Jiaotong University. The detailed clinical features of the Ren Ji cohort are listed in **Supplementary Table 1**. Tumor staging was assessed according to the 8th edition staging classification system (24). Prognostic information of these CCA patients was collected every 2–3 months during the first 2 years and then every 3–6 months until May 2019. The RFS was calculated from the date of tumor resection until the detection of tumor relapse, death from a cause other than CCA, or the last follow-up visit.

### Establishment of the LASSO Regression Model

For these 151 candidate mRNAs, the R package “pROC” was used to plot ROC curves. The optimal cutoff value of each mRNA was generated based on the receiver operating characteristic (ROC) curve, and the area under the curve (AUC), sensitivities, and specificities of these mRNAs were also obtained. Ultimately, 93 mRNAs with AUC > 0.55 were utilized to construct the LASSO Cox regression model. According to the cutoff value, 36 patients in the TCGA database were classified into high- or low-expression status according to each mRNA. Based on the expression status data of these 93 DEGs, the R package “glmnet” was used to construct the LASSO Cox regression model. A sequence of lambdas (*λ*s) and models were returned for us, and the best model with the smallest mean cross-validation error was picked out after 100 times of 10-fold cross-validation. Finally, the risk score for each patient was calculated by a linear combination of selected variables, which were weighted by their corresponding coefficients.

### Quantitative Real-Time PCR

Total RNA was extracted and reversed using the RNeasy Mini Kit (Qiagen, Valencia, CA) and the Revert Aid First Strand cDNA Synthesis Kit (Thermo Scientific, Rockford, IL), respectively. The expression of RORA, CNTFR, COLEC10, TNFSF15, SRC, PDGFD, TUBB3, PLXNB3, and 18S mRNA was measured by qRT-PCR using SYBR Green PCR Master Mix, and Ct value was enrolled for data analysis. Related primer sequences are listed in **Supplementary Table 2**. All these experiments were conducted according to the manufacturer’s instructions. All the genetic testing was retrospective.

### Statistical analysis

TIDE score between groups was compared by the Wilcoxon test. Kaplan–Meier (KM) survival, univariate survival, and multivariate survival analyses were performed using the log-rank test and Cox regression model. Time-dependent ROC curves were performed using the R package “pROC”. A two-sided *p*-value < 0.05 was considered significant.

## Results

### Identification of Immune-Related Genes in Cholangiocarcinoma From Public Datasets

**Figure 1:**
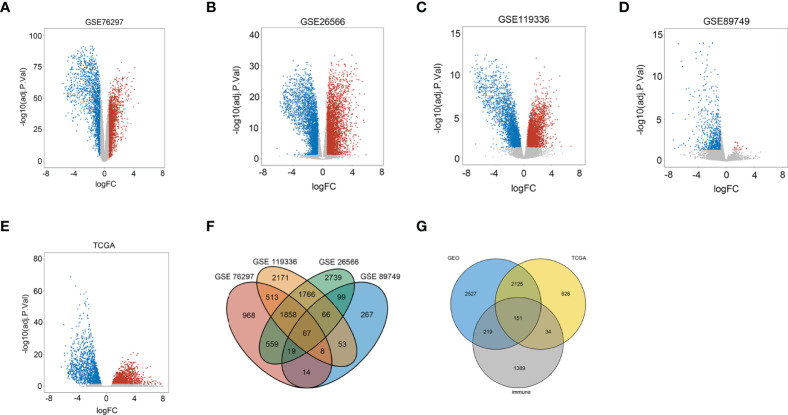
Identification of immune-related differentially expressed genes in CCA from the dataset. **(A)** Volcano plots of DEGs in the GSE76297 dataset. **(B)** Volcano plots of DEGs in the GSE26566 dataset. **(C)** Volcano plots of DEGs in the GSE119336 dataset. **(D)** Volcano plots of DEGs in the GSE89749 dataset. **(E)** Volcano plots of DEGs in the TCGA dataset [*x*-axis: log2(FC); *y*-axis: −log10(FDR) for each gene. Genes with FDR < 0.01 and FC >1.5 or <−1.5 were considered as DEGs in TCGA. Blue: downregulated genes; Gray: non-differential genes; Red: upregulated genes]. **(F)** Overlapping analyses of DEGs in GSE76297, GSE26566, GSE119336, and GSE89749 groups; DEGs shared within 2 datasets or more were regarded as credible DEGs in each Venn diagram. **(G)** Overlapping analysis of GEO, TCGA, and ImmPort datasets.

Genes with adjusted *p*-value < 0.05 and |log2(fold change)| > 0.585 were shown in volcano plots, in which upregulated genes were shown in red and downregulated genes were shown in blue (**Figures 1A–E**). In the four databases, GSE76297, GSE26566, GSE119336, and GSE89749, 2 series or more shared DEGs were regarded as credible DEGs in the GEO Venn diagram, and 5,022 DEGs remained (**Figure 1F**). Then, immune mRNAs were collected from the ImmPort database. The overlapping analysis was further performed among GEO DEGs, TCGA DEGs, and immune mRNAs, and 151 immune-related DEGs (IRDEGs) were identified, which were believed to be commonly dysregulated in CCA (**Figure 1G**).

Next, the R package “pROC” was performed for IRDEGs screening, and 93 genes with AUC ≥ 0.55 remained. GO and KEGG pathway enrichment analyses were performed on the 93 IRDEGs, as shown in (**Supplementary Figures 1A, B**). Most IRDEGs were enriched in platelet degranulation, acute-phase response, and inflammatory regulation. At the same time, most IRDEGs were involved in cytokine–cytokine receptor interaction, JAK-STAT signaling, and the neuroactive ligand–receptor interaction pathway.

### Construction of the Eight-IRDEGs Signature

**Figure 2:**
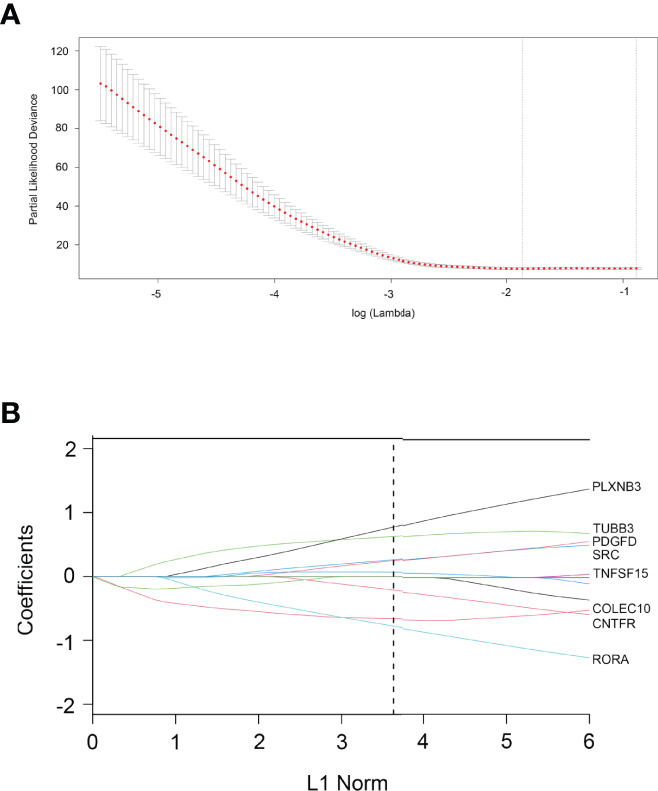
Construction of an 8-IRDEGs signature from the TCGA cohort. **(A)** Tenfold cross-validation for tuning parameter selection in the LASSO model. The dotted vertical lines are drawn at the optimal values by minimum criteria (lambda. min, left vertical dotted line) and 1-SE criteria (lambda.1se, right vertical dotted line). **(B)** LASSO model at optimal lambda value; 8 mRNAs with non-zero coefficients were selected.

According to the 93 IRDEGs cutoff value of the ROC curve, 36 patients were classified into high or low expression status. The “glmnet” package [13, 20] returned a sequence of models (**Supplementary Figure 2A**), and 10-fold cross-validations were performed to select the best one. As shown in **Figure 2A**, a value of *λ* = 0.1624 with log (*λ*) = −0.78937 was chosen by 10-fold cross-validation *via* minimum criteria. However, at different analysis times, the results of the *λ* value might be slightly variable. Therefore, 10-fold cross-validation was run up to 100 times, and the cross-validated errors were averaged. Finally, the *λ* with the smallest mean cross-validation error still returned about 0.1624. At this *λ* value, 8 IRDEGs with non-zero coefficients were selected, including RORA, CNTFR, COLEC10, TNFSF15, SRC, PDGFD, TUBB3, and PLXNB3 (**Figure 2B**). Among them, TNFSF15, SRC, PDGFD, TUBB3, and PLXNB3 were upregulated in CCA, while RORA, CNTFR, and COLEC10 were downregulated. Based on the expression status of these 8 mRNAs, a risk score formula for RFS was constructed as follows: Risk score = (−0.77589 × expression status of RORA) + (−0.65918 × expression status of CNTFR) + (−0.21226 × expression status of COLEC10) + (0.06744 × expression status of TNFSF15) + (0.24810 × expression status of SRC) + (0.26187 × expression status of PDGFD) + (0.62632 × expression status of TUBB3) + (0.77344 × expression status of PLXNB3). In the formula, low expression status was equivalent to 0, and high expression status was equivalent to 1.

### The Correlation Between 8-IRDEGs and Immune Cells

**Figure 3:**
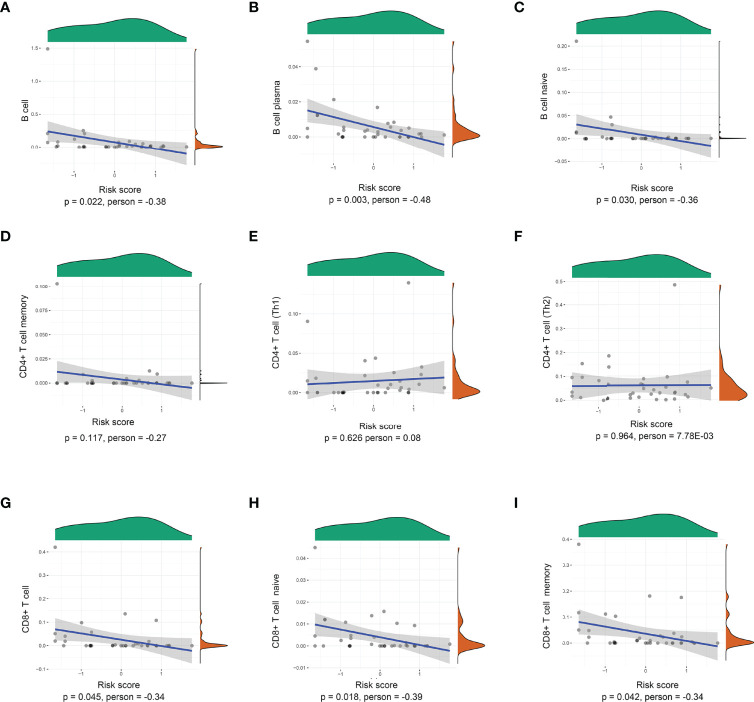
Correlations between the prognostic signature-derived risk score and infiltration abundances of multiple immune cells. **(A)** B cells, **(B)** B-cell plasma, **(C)** B-cell naïve, **(D)** CD4+ T-cell memory, **(E)** CD4+ T cell (Th1), **(F)** CD4+ T cell (Th2), **(G)** CD8+ T cell, **(H)** CD8+ T-cell naïve, and **(I)** CD8+ T-cell memory (Pearson correlation analysis).

The risk scores of recurrence were calculated for each patient in the TCGA cohort. Through the xCell database (25), the expression of 26 immune cells including B cells, CD8+ T cells, and CD4+ T cells was collected. As shown in **Figure 3**, the risk score level was negatively correlated with adaptive immune cells. The lower density of B cell, plasma, B-cell naïve, CD8+ T cell, CD8+ T-cell naïve, and CD8+ T-cell memory was identified in the high-risk subgroup. However, there was no significant relationship between the risk score and CD4+ Th1, CD4+ Th2, and CD4+ T-cell naïve.

Furthermore, the correlation of the 8-IRDEGs with innate immune cells and other scores is shown in **Supplementary Figure 3**. The risk score was not significantly correlated with endothelial cells, macrophages, monocytes, neutrophils, and NK cells.

Furthermore, a significant negative correlation was found between the stroma score (Pearson correlation analysis, *p* = 0.028), immune score (Pearson correlation analysis, *p* = 0.010), ESTIMATEScore (Pearson correlation analysis, *p* = 0.009), and the 8-IRDEGs (**Supplementary Figures 4A, C, E**). Meanwhile, patients with lower risk scores displayed a higher stroma score (Fisher’s exact test, *p* = 0.015), immune score (Fisher’s exact test, *p* = 0.054), and ESTIMATEScore (Fisher’s exact test, *p* = 0.015) (**Supplementary Figures 4B, D, F**).

### Evaluation of the Risk Score Formula for Recurrence in the TCGA Cohort

**Figure 4:**
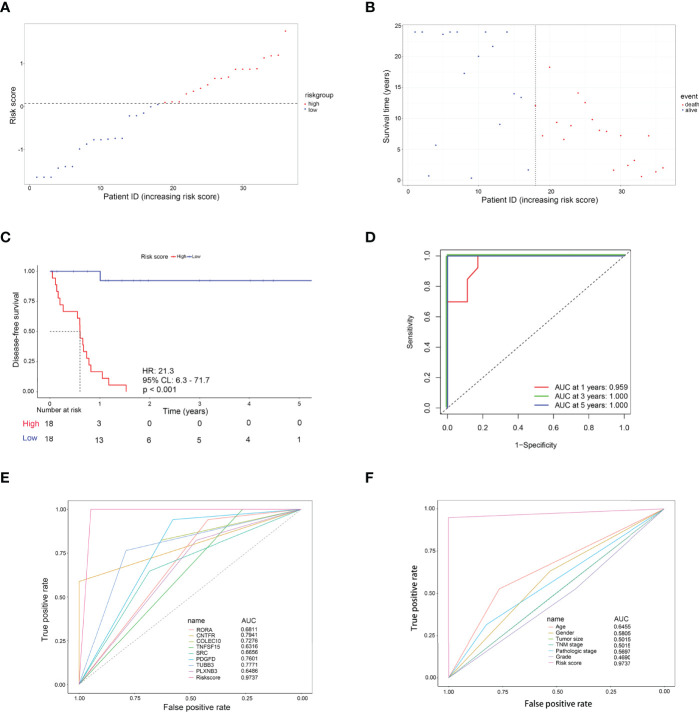
Evaluation of the 8-IRDEGs signature for relapse in the TCGA cohort. **(A)** Distribution of the risk score derived from the signature. Patients are ranked according to the corresponding risk score. **(B)** Survival status of CCA patients in different risk subgroups. **(C)** The Kaplan–Meier survival curve of recurrence-free for patients between two different groups. **(D)** Time-dependent ROC curve at 1, 3, and 5 years. **(E)** Comparison of prognostic accuracy between the signature and single mRNAs. **(F)** Comparison of prognostic accuracy between the signature and clinical characteristics. *p*-values were calculated using the log-rank test. HR, hazard ratio; AUC, area under the ROC curve; RFS, recurrence-free survival.

As shown in **Figure 4A**, with the median risk score as the cutoff value, all CCA patients were assigned to either a high-risk or a low-risk group (**Figure 4A**). Also, an overview of the DFS and IRDEGs expression of these groups is shown in **Figure 4B**. KM analysis demonstrated that CCA patients with higher risk scores had significantly worse RFS than those with lower risk scores (HR = 21.3, 95% CI: 6.3–71.7, *p* < 0.001, **Figure 4C**). Moreover, the time-dependent ROC curves between the 8-IRDEGs signature and RFS showed that the 1-year, 3-year, and 5-year AUC were 0.959, 1.000, and 1.000, respectively (**Figure 4D**). In addition, compared with any single gene or clinical feature, the 8-IRDEGs signature had a favorable recurrence predictive value (**Figures 4E, F**).

Furthermore, KM analysis showed that each IRDEG was tightly associated with the DFS of CCA patients (all *p* < 0.05) (**Supplementary Figure 5**). Univariate survival analysis verified that the 8-IRDEGs signature also had a better prognostic value than each immune-related gene and many clinical factors (**Supplementary Figure 6**). However, clinical association analyses showed that increased risk score was not related to the clinical characteristics (**Supplementary Table 3**).

To further investigate the applicable CCA population of this 8-IRDEGs signature, the 8-IRDEGs signature-based survival analyses were performed in subgroups of patients with different clinical variables. The 8-IRDEGs prognostic risk index consistently stratified patient survival regardless of gender (*p* = 0.0139 in male patients; *p* = 4e-04 in female patients), age (*p* = 0.0139 in less than or equal to 60 years; *p* = 4e-04 in over 60 years), tumor size (*p* = 8e-04 in T1; *p* = 0.0022 in T2+T3+T4), stages (*p* = 8e-04 in I; *p* = 8e-04 in II+III+IV), and grade (*p* = 0.0023 in grade I; *p* = 0.023 in grade II+III). Furthermore, the significant difference was shown in patients with pathologic stage I+II (*p* = 1e-04). However, because of the small sample size, patients with pathologic stage III+IV were a little powerless for relapse prediction (*p* = 0.0588) (**Supplementary Figure 7**).

### Molecular Characteristics of Different 8-IRDEGs Signature Subgroups

**Figure 5:**
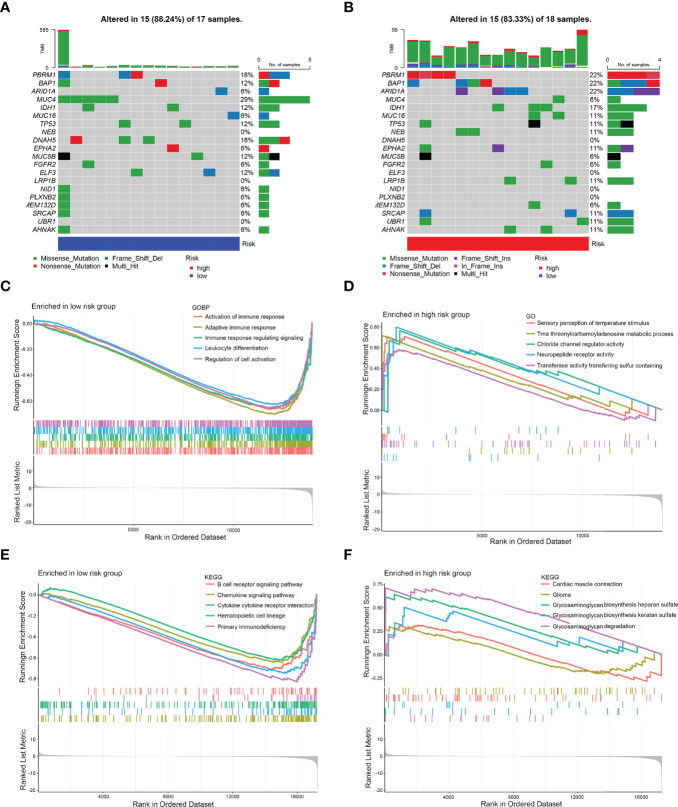
Mutation analysis and GSEA. **(A)** Significantly mutated genes in the mutated CCA samples of the low-risk subgroup. **(B)** Significantly mutated genes in the mutated CCA samples of the high-risk subgroup. [Samples (columns) are arranged to emphasize mutual exclusivity among mutations. The right panel shows the mutation percentage, and the top panel shows the overall number of mutations. The color coding indicates the mutation type.] **(C)** Gene sets enriched in the low-risk subgroup (*p* < 0.05). **(D)** Gene sets enriched in the high-risk subgroup (*p* < 0.05). **(E)** KEGG pathway in the low-risk subgroup (*p* < 0.05). **(F)** KEGG pathway in the high-risk subgroup (*p* < 0.1).

Then, gene mutations enabled us to gain further biological insight into the immunological properties of the 8-IRDEGs signature subgroups. A missense mutation was the most common mutation type, followed by nonsense and frameshift deletions. The mutation rates of MUC4, PBRM1, DNAH5, BAP1, IDH1, TP53, MUC5B, ARID1A, ELF3, MUC16, NEB, EPHA2, LRP1B, SRCAP, UBR1, and AHNAK were higher than 10% in both groups. Mutations in MUC4, PBRM1, and DNAH5 genes were more common in the low-risk subgroup (**Figure 5A**), while mutations in PBRM1, BAP1, and ARID1A genes were more common in the high-risk subgroup (**Figure 5B**).

Gene Set Enrichment Analysis (GSEA) was performed to determine the gene sets enriched in different subgroups. The gene sets of the low-risk samples were enriched in immune response activation (**Figure 5C**) (*p* < 0.05). While the gene sets of the high-risk samples were enriched in sensory perception of temperature stimulus (**Figure 5D**) (*p* < 0.05). Moreover, the low-risk subgroup was involved in the B-cell receptor signaling pathway (**Figure 5E**) (*p* < 0.05); however, the high-risk subgroup was involved in myocardial contraction (**Figure 5F**) (*p* < 0.5), which may be attributed to the small number of samples.

### Immune Characteristics of the Two 8-IRDEGs Signature Subgroups

**Figure 6:**
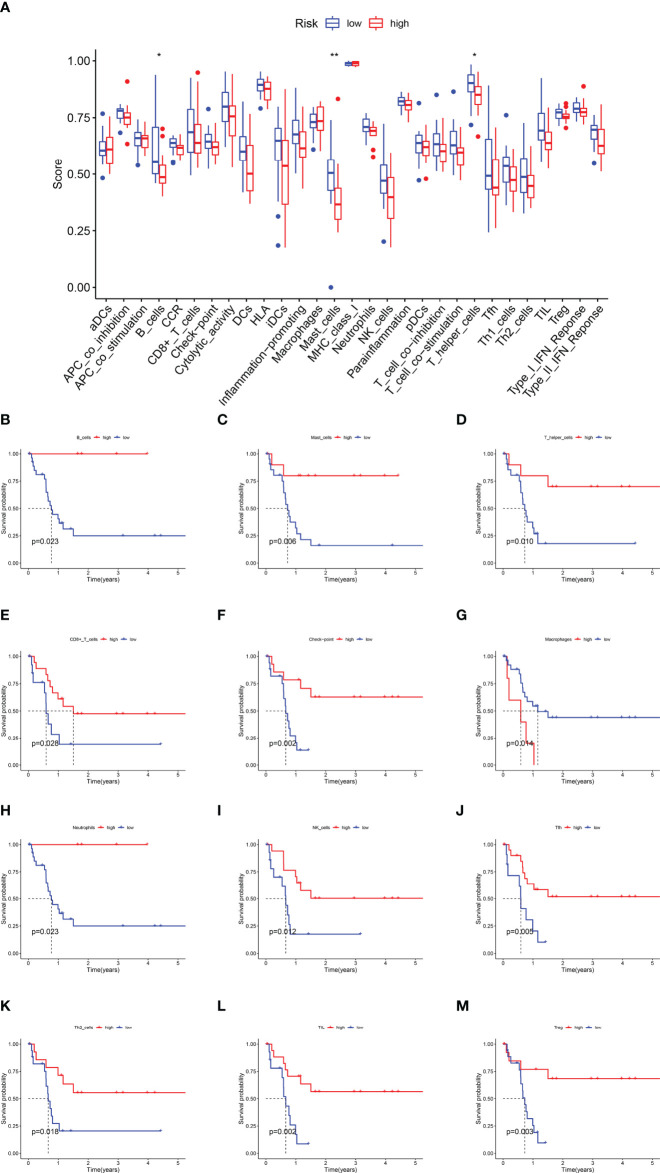
The Kaplan–Meier survival analysis for immune functions. **(A)** The difference of immune functions between the high-risk and low-risk subgroups. **(B)** The KM curve of B cells. **(C)** The KM curve of Mast cells. **(D)** The KM curve of T helper cells. **(E)** The KM curve of CD8+T cells. **(F)** The KM curve of the checkpoint. **(G)** The KM curve of Macrophages. **(H)** The KM curve of Neutrophils. **(I)** The KM curve of NK cells. **(J)** The KM curve of Tfh cells. **(K)** The KM curve of Th2 cells. **(L)** The KM curve of TIL. **(M)** The KM curve of Treg (*p < 0.05, ** *p* < 0.01).

Immune functions, such as those of B cells, mast cells, and T-helper cells, were significantly suppressed in the high-risk subgroup (*p* < 0.05) (**Figure 6A**). KM survival curves exhibited that the activity of immune function could predict the DFS of CCA patients. The more active the immune function, the lower the tumor recurrence rate (*p* < 0.05) (**Figures 6B–L**, **Supplementary Figure 8**), while CCA tended to relapse more in the higher activity of the macrophage subgroup (*p* = 0.014) (**Figure 6M**).

TIDE was used to assess the potential clinical efficacy of immunotherapy in different risk subgroups. A higher TIDE prediction score represented a higher likelihood of immune evasion, indicating that the patients were less likely to benefit from ICI therapy. However, there was no difference in TIDE, MSI, dysfunction, and exclusion between high- and low-risk subgroups (**Supplementary Figures 9A–D**). Next, the relationship between the risk score and PD-L1 expression and TMB is shown in **Supplementary Figures 9E–H**, while the risk score was not significantly correlated with PD-L1 and TMB. Interestingly, the AUC for the 8-IRDEGs signature was better than TIS and TIDE at 1, 3, and 5 years of follow-up (**Supplementary Figures 9I–K**).

### Validation of the 8-IRDEGs Signature for Relapse Prediction in the Ren Ji Cohort

**Figure 7:**
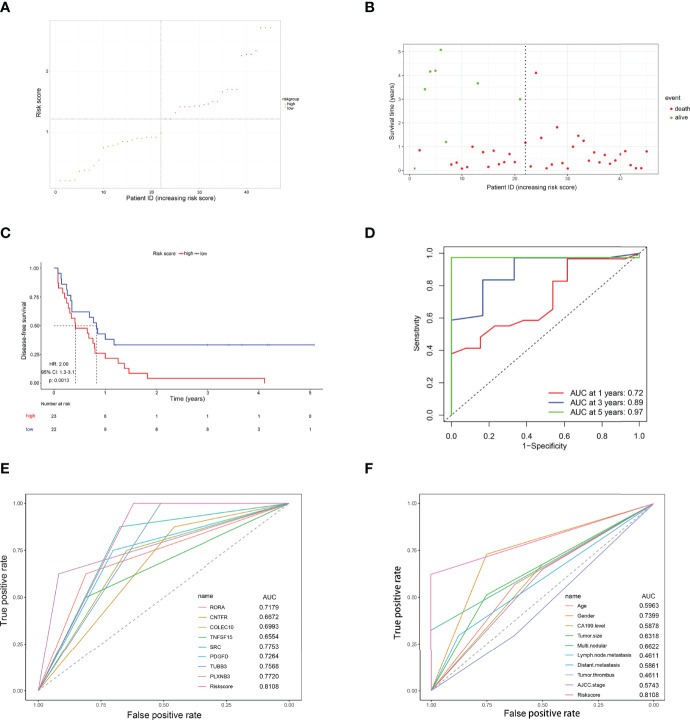
Evaluation of the risk score formula for relapse prediction in the TCGA cohort. **(A)** Scatter plot for the distribution of risk score and relapse status of individual patients. **(B)** Survival status of CCA patients in the two 8-IRDEGs signature subgroups. **(C)** The Kaplan–Meier survival curve of recurrence-free for patients between two different groups. **(D)** Time-dependent ROC curve at 1, 3, and 5 years. **(E)** Comparison of prognostic accuracy between the signature and single mRNAs. **(F)** Comparison of prognostic accuracy between the signature and clinical characteristics. *p*-values were calculated using the log-rank test. HR, hazard ratio; AUC, area under the ROC curve; RFS, recurrence-free survival.

To further verify whether this 8-IRDEGs classifier had a similar predictive ability in different CCA populations, it was applied to the Ren Ji Hospital cohort. According to the median risk score determined by the ROC curve, patients were further divided into high-risk (*n* = 23) or low-risk (*n* = 22) groups. As shown in **Figures 7A, B**, as the risk score increased, CCA was more likely to relapse after resection. Survival analysis showed that patients in the high-risk group had shorter RFS time than those in the low-risk group (*p* = 0.0013, HR = 2.00, 95% CI 1.30–3.10, **Figure 7C**). The AUCs of the time-dependent ROC curves between the 8-IRDEGs signature and RFS were 0.720 for 1 year, 0.890 for 3 years, and 0.970 for 5 years (**Figure 7D**). Moreover, the AUC of the 8-IRDEGs signature was significantly greater than any immune-related gene or clinical characteristics (**Figures 7E, F**). Univariable Cox analyses of the Ren Ji cohort showed that the 8-IRDEGs signature was a significant factor related to the RFS of CCA (**Supplementary Figure 10**). Unfortunately, clinical association analyses showed that the increased risk score was not associated with the clinical characteristics (**Supplementary Table 4**).

At the same time, this 8-IRDEGs signature was a perfect predictor that was independent of some clinicopathological characteristics like age, tumor size, and tumor thrombus in the Renji cohort (**Supplementary Figure 11**). For patients in the following subgroups: female, negative lymph node metastasis or distant metastasis, mono-modular, CA19-9 ≤ 37 ng/ml, and stage I, the immune model maintained its predictive value for disease-free survival. Unfortunately, the model lost its prognostic role for patients in the following subgroups: male, CA19-9 > 37 ng/ml, multi-modular, positive lymph node metastasis or positive distant metastasis, multi-modular, and stage II+III+IV, which might be due to the small sample size of these subgroups.

## Discussion

Immune infiltration in the tumor environment is actively involved in the progression of many solid tumors, including CCA. Accumulating evidence highlights that the response to antitumor therapy and the DFS of CCA patients is subject to host immunity. In this regard, an immune-based prognostic signature can be rationally applied to identify patients with recurrence in advance.

In this study, immune-related DEGs were selected from GEO and TCGA, and the LASSO model was used to establish the 8-IRDEGs signature to predict CCA relapse after liver resection. Compared with each immune-related gene and clinical factor, the 8-IRDEGs signature had a better predictive value. In the two independent cohorts, univariate survival analysis demonstrated that the 8-IRDEGs signature was tightly associated with DFS, which could be an independent risk factor. In summary, the model contributed to the individualized treatment and management of CCA patients after surgery.

The 8-IRDEGs signature was composed of RORA, CNTFR, COLEC10, TNFSF15, SRC, PDGFD, TUBB3, and PLXNB3, and each gene played a vital role in tumor immunity. RORA/C transcription factors had been confirmed to augment tumor growth and cell proliferation in non-small cell lung cancer (26). CNTFR, as a ciliary neurotrophic receptor, is combined with CLCF1 to stimulate B-cell differentiation and antibody production (27). Zhang et al. (28) found that decreased expression of COLEC10 might predict poorer overall survival in HCC patients; however, the association between COLEC10 and tumor immunity has not been reported. TNFSF15(TL1A) is a tumor necrosis factor (TNF) family member expressed by monocytes, macrophages, and other immune cells, while TNFSF15/DR3 pathways might represent an effective therapeutic target for chronic immunological diseases (29, 30). To a lesser degree, TL1A increased the lysis of colorectal adenocarcinoma epithelial-derived lines by IL-12/IL-18-activated cells (31). Therefore, TNFSF15 might improve the tumor progression to a certain extent. Xiao et al. found that protein tyrosine phosphatase 2 containing the Src homology 2 domain dampened T cell-mediated antitumor immunity by restraining the macrophage/CXCL9-T cell/IFN-γ feedback loop (32). PDGFD signaling in GBM was shown to induce IFN-γ secretion by natural killer cells through the engagement of the human immunoreceptor NKp44 (33). Meanwhile, PDGFD was an important predictor gene for bladder cancer, renal clear cell carcinoma, and osteosarcoma (34–36). It has been found that TUBB3 was highly expressed in lung neuroendocrine carcinoma and medulloblastoma, which was associated with positive lymphatic permeation (37, 38). In the majority of cancers, such as CCA and prostate adenocarcinoma, PLXNB3 was more associated with poor survival (39). In summary, the association between CCA and immunity needed more research.

Furthermore, this immune signature was negatively relevant to infiltration in the tumor microenvironment, especially adaptive immunity. A preponderance of CD8+ T cells and CD4+ T cells at the tumor–liver interface was related to longer overall survival and the presence of tumor-infiltrating CD4+ or CD8+ T cells (40–42). Similarly, the presence of B cells predicted a favorable prognosis in CCA (40). Therefore, in our research, the density of adaptive immune cells was decreased in the high-risk subgroup. Moreover, KM curve analysis has shown that the adaptive immune function predicted a better prognosis in this paper. NK cells and M2-like macrophage cells were associated with poor outcomes as immunosuppressive cells (43, 44). Consistently, dense infiltration of macrophages indicated a poor prognosis in **Figure 6G**, while the relationship between NK cells and prognosis has been verified in cell lines and mouse xenograft models. The NK cells in our study could reduce the CCA recurrence; however, more research is needed to further confirm the finding. Unfortunately, there was no obvious correlation between the risk score and innate immune cells, which was due to the lack of uniformity in the assays, the small number of samples, and the variability of the thresholds used to define immune cell infiltration. Furthermore, a significant negative correlation was found between the stroma score, immune score, ESTIMATEScore, and risk score, which might be beneficial for predicting individual tumor microenvironment. These findings revealed that the immune-related signature could be applied to provide immunotherapy targets for CCA patients.

Regarding genomic alterations, CCA falls midway in the cancer mutational spectrum, and gene mutations are associated with poor prognosis (45, 46), for example, IDH, EPHA2, BRAF, BAP1 mutations, and FGFR2 fusions in intrahepatic CCA, whereas extrahepatic tumors specifically show PRKACA, PRKACB, ELF3, and ARID1B mutations. In this regard, mutation analysis was shown in two 8-IRDEGs signature subgroups (14, 47–49). Gene mutations of PBRM1, BAP1, and ARID1A were more common in the high-risk subgroup, and one of the three chromatin-remodeling genes trended toward worse survival compared with subjects whose all three genes were wild type (3-year survival of 47.1% for subjects with mutations compared with 93.3% for subjects without mutations) (50). On the other hand, MUC4, PBRM1, and DNAH5 genes were more common in the low-risk subgroup. Among them, the positive or high expression level of MUC4 was significantly related to poor survival in patients after CCA resection (51). In the future, different targeted drugs can be used in different risk subgroups.

Recently, two distinct tumor immune evasion mechanisms have been discovered (52, 53). Some tumors have a high level of cytotoxic T-cell infiltration, but these T cells tend to be in a dysfunctional state. In other tumors, immunosuppressive factors may exclude T cells from infiltrating tumors (54). Therefore, TIDE was developed to identify factors that underlie these two tumor immune escape mechanisms. On the other hand, microsatellite instability (MSI) is considered a potentially meaningful predictive biomarker of the response to ICIs. However, dysfunction, exclusion, MSI, and TIDE did not differ significantly between the high- and low-risk subgroups. The reasons might be as follows: one is that the small number of samples could not fully magnify the difference; the other is that T-cell dysfunction scores were computed in different cancer datasets, including TCGA, PRECOG17, and METABRIC32 databases. Nevertheless, the samples in our study were only from the TCGA database, which resulted in bias (55).

The expression of PD-L1 assessed by immunohistochemistry has been shown to correlate with the response to ICIs in several tumor types, and Gani and colleagues evaluated that iCCAs expressing PD-L1 in the TF had a 60% reduced survival compared with PD-L1-negative patients (56). In addition to PD-L1 expression, TMB has also been associated with the response to ICIs in several tumor types (57). Unfortunately, the relationship between TME, PD-L1 (CD274), and risk score was not close. As we all know, PD-L1 and TMB assessment is widely influenced by the kits and methods used, and these kits and methods have been suggested to report different values in the same sample. Therefore, the difference was not shown in our research (58, 59). Furthermore, we found that the 8-IRDEGs signature had better prediction than TIDE and TIS at 1, 3, and 5 years, respectively, which might be a prognostic marker for immunotherapy.

Currently, the ICI pembrolizumab is verified in patients with microsatellite-instable tumors (60). Huang et al. found that the mRNA vaccine such as CD247, FCGR1A, and TRRAP could benefit patients with IS2 tumors (immunologically quiet or TGF-β dominant) (61). However, the clinical data on immune-directed therapies in CCA are still limited. Besides immune cells, there are many other cells such as cancer-associated fibroblasts (CAFs) in the CCA microenvironment, which also contribute to CCA progression. Therefore, target CAFs may be another CAF treatment option. Furthermore, the abundant extracellular matrix can prevent drug entry, which may be another aspect we need to explore. In the future, CCA immunotherapy must target not only immune cells but also other major cells within the stroma of CCA (1). In our study, the 8-IRDEGs signature we constructed can reflect immune cell infiltration in CCA patients and help clinicians to make a personalized diagnosis and immunotherapy plans, which can avoid unnecessary waste of medical resources.

Finally, there were some limitations to this model. Since the four GEO datasets involved in this study may not include all of the possible mRNA present, the mRNA candidates identified in the model may not represent the complete mRNA populations underlying CCA biological behavior. Secondly, immune infiltrating cells were not evaluated in the clinical fresh patient samples, and more fresh samples should be included to clarify the role of this model in predicting immune cell infiltration. Finally, the mechanism behind the signature mRNAs should also be explored in future research; in addition, the cellular function and molecular mechanism of the 8-IRDEGs needed to be further explored by experimental studies.

## Conclusion

The 8-IRDEGs signature was constructed from GEO and TCGA databases and was verified in the Renji cohort; it has several potential clinical applications: firstly, it may be used to predict the progression of an individual CCA patient. Then, GSEA showed that the model might involve a variety of cancer recurrence and metastasis-associated pathways, which supported the RFS predictive ability of the signature. Next, the signature could reveal different gene mutations in low- or high-risk groups, which might contribute to targeted therapy. Most importantly, the 8-IRDEGs signature could reflect immune cell infiltration in each CCA patient and help clinicians make personalized immunotherapy plans, which can avoid unnecessary waste of medical resources. In future studies, we and other investigators ought to further validate the efficiency of this model designed for clinical trials, including predicting prognosis, individualized assessment of immune status and genetic mutations, and improving a personalized therapeutic schedule.

## Data Availability Statement

The datasets presented in this study can be found in online repositories. The names of the repository/repositories and accession number(s) can be found in the article/**Supplementary Material**.

## Ethics Statement

The studies involving human participants were reviewed and approved by the Ethics Review Committee of Renji Hospital, School of Medicine, Shanghai Jiaotong University. The patients/participants provided their written informed consent to participate in this study.

## Author Contributions

HG had the idea and drafted the manuscript. YQ and YY analyzed the data and prepared the figures and tables. YB and JJ provided clinical information and statistical advice. HJ and CY prepared clinical samples, extracted RNA, and performed qRT-PCR assays. HW, YS, and XK supervised the study and revised the manuscript. All authors contributed to the article and approved the submitted version.

## Funding

This work was supported by the National Natural Science Foundation of China (82070633 to XK, 31870905 to HW), the Program of Shanghai Academic/Technology Research Leader (20XD1403700) to XK, the Zhejiang Provincial Program for the Cultivation of High-level Innovative Health Talents to YS.

## Conflict of Interest

The authors declare that the research was conducted in the absence of any commercial or financial relationships that could be construed as a potential conflict of interest.

## Publisher’s Note

All claims expressed in this article are solely those of the authors and do not necessarily represent those of their affiliated organizations, or those of the publisher, the editors and the reviewers. Any product that may be evaluated in this article, or claim that may be made by its manufacturer, is not guaranteed or endorsed by the publisher.

## Supplementary Material

Supplementary Figure 1
**(A)** Enriched Gene Ontology terms including biological process, cellular component, and molecular function. **(B)** Enriched KEGG pathways.

Supplementary Figure 2 Complete LASSO coefficient profiles of the 93 mRNAs. Each curve represents a variable. On the above axis: the number of nonzero coefficients at λ varies. X-axis: L1 Norm, the summation of absolute nonzero coefficients at as λ varies. Y-axis: the values of nonzero coefficients at as λ varies.

Supplementary Figure 3 Correlations between the prognostic signature-derived risk score and infiltration abundances of multiple immune cells. **(A)** Endothelial cell, **(B)** Macrophage, **(C)** Monocyte, **(D)** Neutrophil, **(E)** NK cell. (person correlation analysis).

Supplementary Figure 4 8-mRNAs are significantly negatively correlated with TME. **(A)** Scatter plots depicting the negative correlation between 8-mRNAs and stroma score (person correlation analysis, p = 0.028). **(B)** The proportion of patients with low/high stroma scores is based on risk score stratification (Fisher’s exact test, p value= 0.015). **(C)** Scatter plots depicting the negative correlation between 8-mRNAs and immune score (person correlation analysis, p =0.010). **(D)** The proportion of patients with low/high immune scores is based on risk score stratification (Fisher’s exact test, p value= 0.054). **(E)** Scatter plots depicting the negative correlation between 8-mRNAs and ESTIMATEScore (person correlation analysis, p =0.009). **(F)** The proportion of patients with low/high ESTIMATEScore is based on risk score stratification (Fisher’s exact test, p value= 0.015).

Supplementary Figure 5 KM curve of 8-mRNAs in TCGA database. **(A)** The Kaplan-Meier survival analysis of the RORA, **(B)** The Kaplan-Meier survival analysis of the CNTFR, **(C)** The Kaplan-Meier survival analysis of the COLEC10, **(D)** The Kaplan-Meier survival analysis of the TNFSF15, **(E)** The Kaplan-Meier survival analysis of the SRC, **(F)** The Kaplan-Meier survival analysis of the PDGFD, **(G)** The Kaplan-Meier survival analysis of the TUBB3, **(H)** The Kaplan-Meier survival analysis of the PLXNB3. (P-values were calculated using the log-rank test. HR, hazard ratio).

Supplementary Figure 6 Univariate survival analysis in the TCGA cohort. **(A)** Univariate survival analysis for single IRDEG and risk score. **(B)** Univariate survival analysis for clinical factor and risk score.

Supplementary Figure 7 Kaplan-Meier survival analyses of the TCGA cohort, according to the 8-IRDEGs-based classifier stratified by clinicopathological characteristics. **(A, B)** Gender, **(C, D)** Age, **(E, F)** Tumor size, **(G, H)** AJCC stage, **(I, J)** Pathologic stage, and **(K, L)** Grade. (P-values were calculated using the log-rank test. HR, hazard ratio).

Supplementary Figure 8 The Kaplan-Meier survival analysis for innate immune functions. **(A)** The KM curve of the aDCs, **(B)** The KM curve of the APC-co-inhibition, **(C)** The KM curve of the CCR, **(D)** The KM curve of the DCs, **(E)** The KM curve of the HLA, **(F)** The KM curve of the iDCs, **(G)** The KM curve of the inflammation-promoting, **(H)** The KM curve of the Parainflammation. **(I)** The KM curve of the pDC, **(J)** The KM curve of the T-cell-co-inhibition, **(K)** The KM curve of the T-cell-co-stimulation, **(L)** The KM curve of the Type-II-IFN-Response. (P-values were calculated using the log-rank test. HR, hazard ratio).

Supplementary Figure 9 The prognostic value of 8-IRDEGs signature in patients with anti-PD-L1 therapy. T cell dysfunction **(A)** and exclusion score **(B)**, MSI **(C)**, and TIDE **(D)** in different risk score subgroups. The score between the two subgroups were compared through the Wilcoxon test (ns: not significant, *p < 0.05, ** p < 0.01, *** p < 0.001). **(E)** TME in different risk score subgroups, **(F)** Correlations between the prognostic signature-derived risk score and TME. **(G)** PD-L1 in different risk score subgroups, **(H)** Correlations between the prognostic signature-derived risk score and PD-L1, **(I–K)** ROC analysis of 8-IRDEGs signature, TIS, and TIDE on DFS at 1- **(I)**, 3- **(J)**, and 5-years **(K)** follow-up. (person correlation analysis, AUC, area under ROC curve).

Supplementary Figure 10 Univariate survival analysis for the clinical factor and risk score in Renji Hospital.

Supplementary Figure 11 Kaplan-Meier survival analyses of the Ren Ji cohort, according to the 8-IRDEGs -based classifier stratified by clinicopathological characteristics. **(A, B)** Gender, **(C, D)** Age, **(E,F)**Tumor size, **(G,H)** Lymph node metastasis, **(I,J)** Modular, **(K, L)** CA 19-9 level, **(M,N)** Distant metastasis,**(O,P)** Tumor thrombus,**(Q,R)** Stage. (P-values were calculated using the log-rank test. HR, hazard ratio).

## Abbreviations

CCA, cholangiocarcinoma; GEO, Gene Expression Omnibus; TCGA, The Cancer Genome Atlas; ImmPort, Immunology Database and Analysis Portal; LASSO, least absolute shrinkage and selection operator; RFS, recurrence-free survival; TIDE, tumor immune dysfunction and exclusion; TIS, tumor inflammation signature; IRDEGs, immune-related differentially expressed genes; pCCA, perihilar cholangiocarcinoma; iCCA, intrahepatic cholangiocarcinoma; ROC, receiver operating characteristic; MSI, microsatellite instability; ICIs, immune checkpoint inhibitors.
