# Supplementary figures and images for: An Immunity-Related Gene Model Predicts Prognosis in Cholangiocarcinoma
Source: Front Oncol. 2022 Jul 1;12:791867. doi: 10.3389/fonc.2022.791867 (PMC9283581; doi:10.3389/fonc.2022.791867)

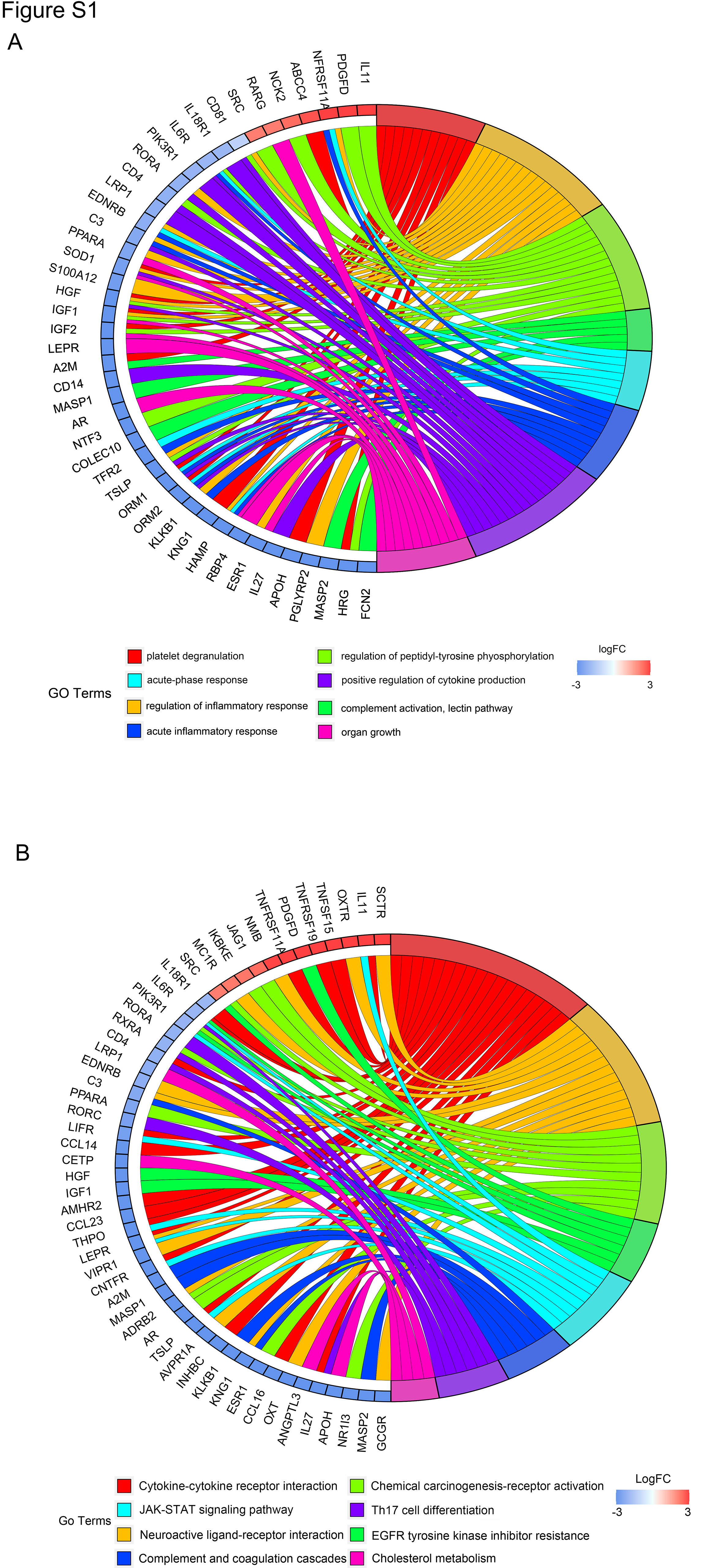

Supplement: Supplementary Figure 1 — (A) Enriched Gene Ontology terms including biological process, cellular component, and molecular function. (B) Enriched KEGG pathways. [file Image_1.tif]

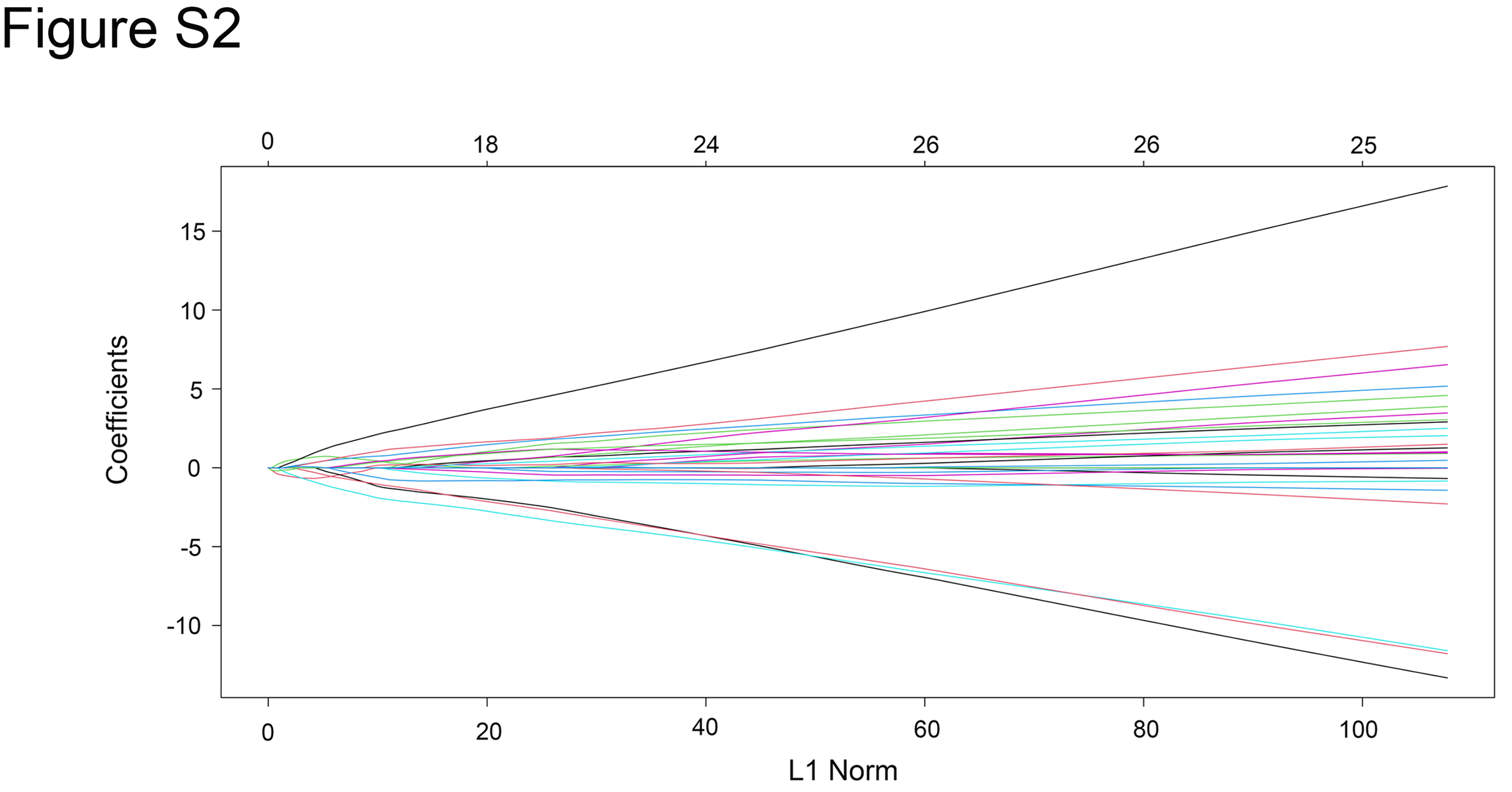

Supplement: Supplementary Figure 2 — Complete LASSO coefficient profiles of the 93 mRNAs. Each curve represents a variable. On the above axis: the number of nonzero coefficients at λ varies. X-axis: L1 Norm, the summation of absolute nonzero coefficients at as λ varies. Y-axis: the values of nonzero coefficients at as λ varies. [file Image_2.tif]

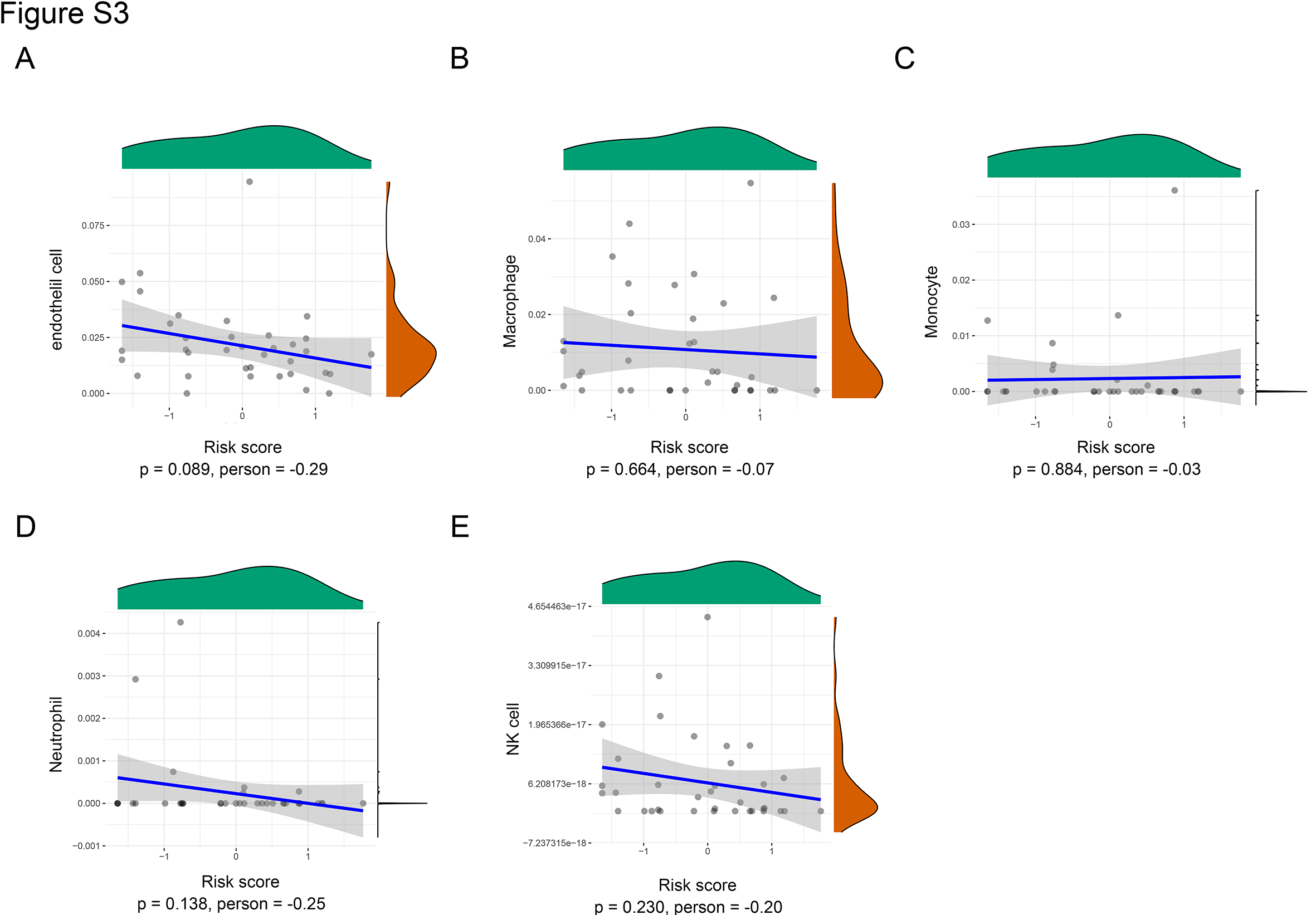

Supplement: Supplementary Figure 3 — Correlations between the prognostic signature-derived risk score and infiltration abundances of multiple immune cells. (A) Endothelial cell, (B) Macrophage, (C) Monocyte, (D) Neutrophil, (E) NK cell. (person correlation analysis). [file Image_3.tif]

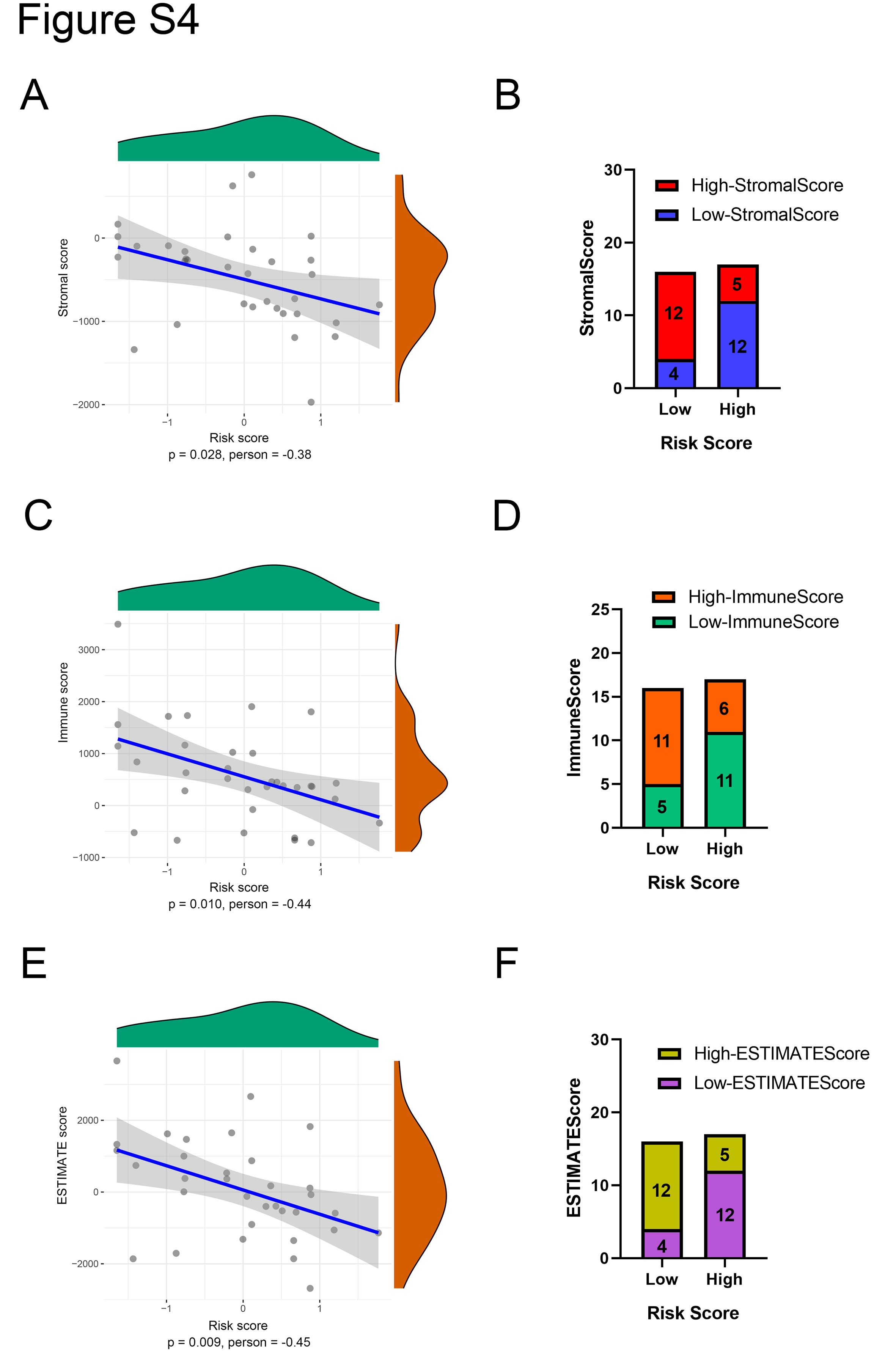

Supplement: Supplementary Figure 4 — 8-mRNAs are significantly negatively correlated with TME. (A) Scatter plots depicting the negative correlation between 8-mRNAs and stroma score (person correlation analysis, p = 0.028). (B) The proportion of patients with low/high stroma scores is based on risk score stratification (Fisher’s exact test, p value= 0.015). (C) Scatter plots depicting the negative correlation between 8-mRNAs and immune score (person correlation analysis, p =0.010). (D) The proportion of patients with low/high immune scores is based on risk score stratification (Fisher’s exact test, p value= 0.054). (E) Scatter plots depicting the negative correlation between 8-mRNAs and ESTIMATEScore (person correlation analysis, p =0.009). (F) The proportion of patients with low/high ESTIMATEScore is based on risk score stratification (Fisher’s exact test, p value= 0.015). [file Image_4.tif]

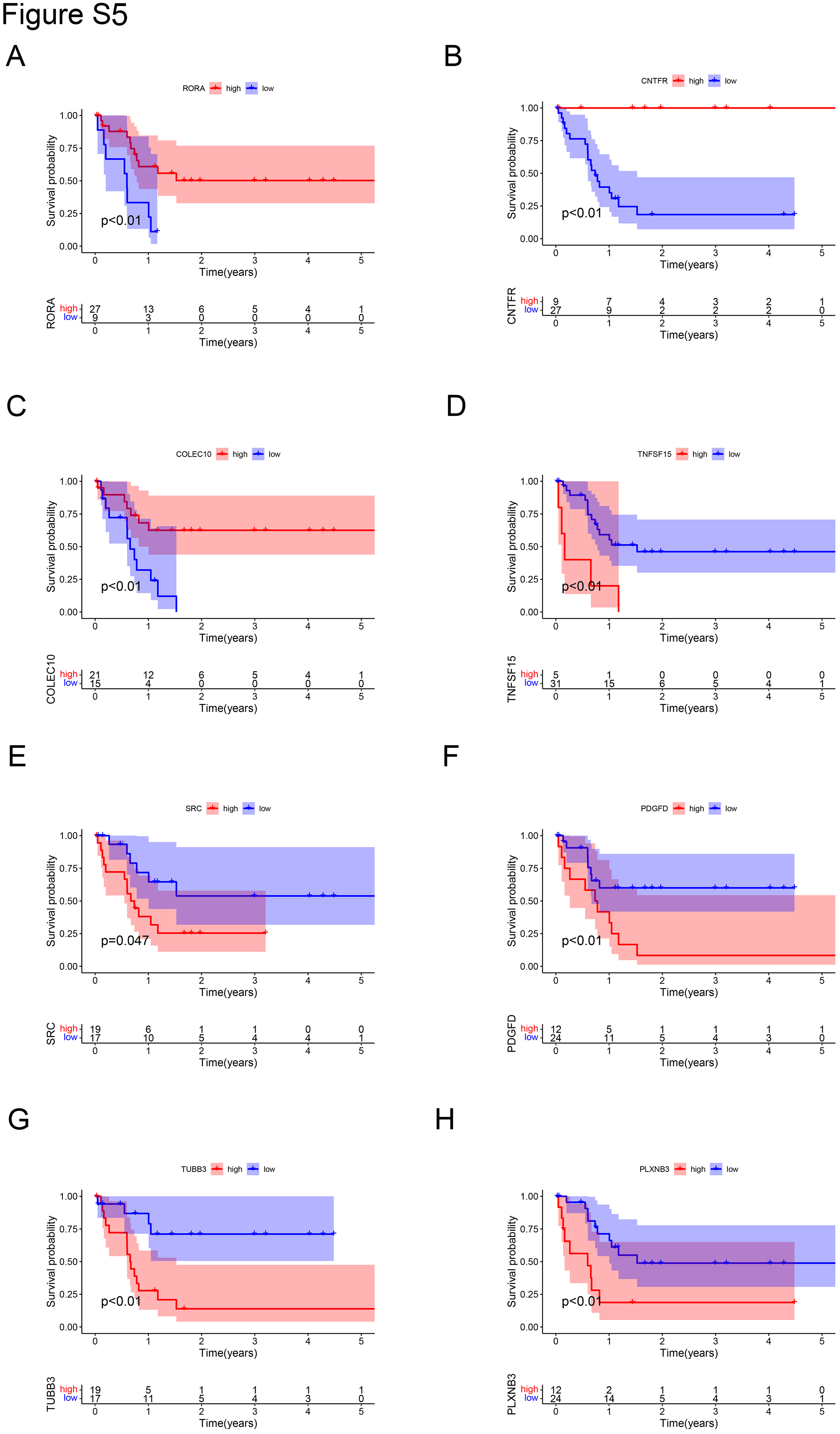

Supplement: Supplementary Figure 5 — KM curve of 8-mRNAs in TCGA database. (A) The Kaplan-Meier survival analysis of the RORA, (B) The Kaplan-Meier survival analysis of the CNTFR, (C) The Kaplan-Meier survival analysis of the COLEC10, (D) The Kaplan-Meier survival analysis of the TNFSF15, (E) The Kaplan-Meier survival analysis of the SRC, (F) The Kaplan-Meier survival analysis of the PDGFD, (G) The Kaplan-Meier survival analysis of the TUBB3, (H) The Kaplan-Meier survival analysis of the PLXNB3. (P-values were calculated using the log-rank test. HR, hazard ratio). [file Image_5.tif]

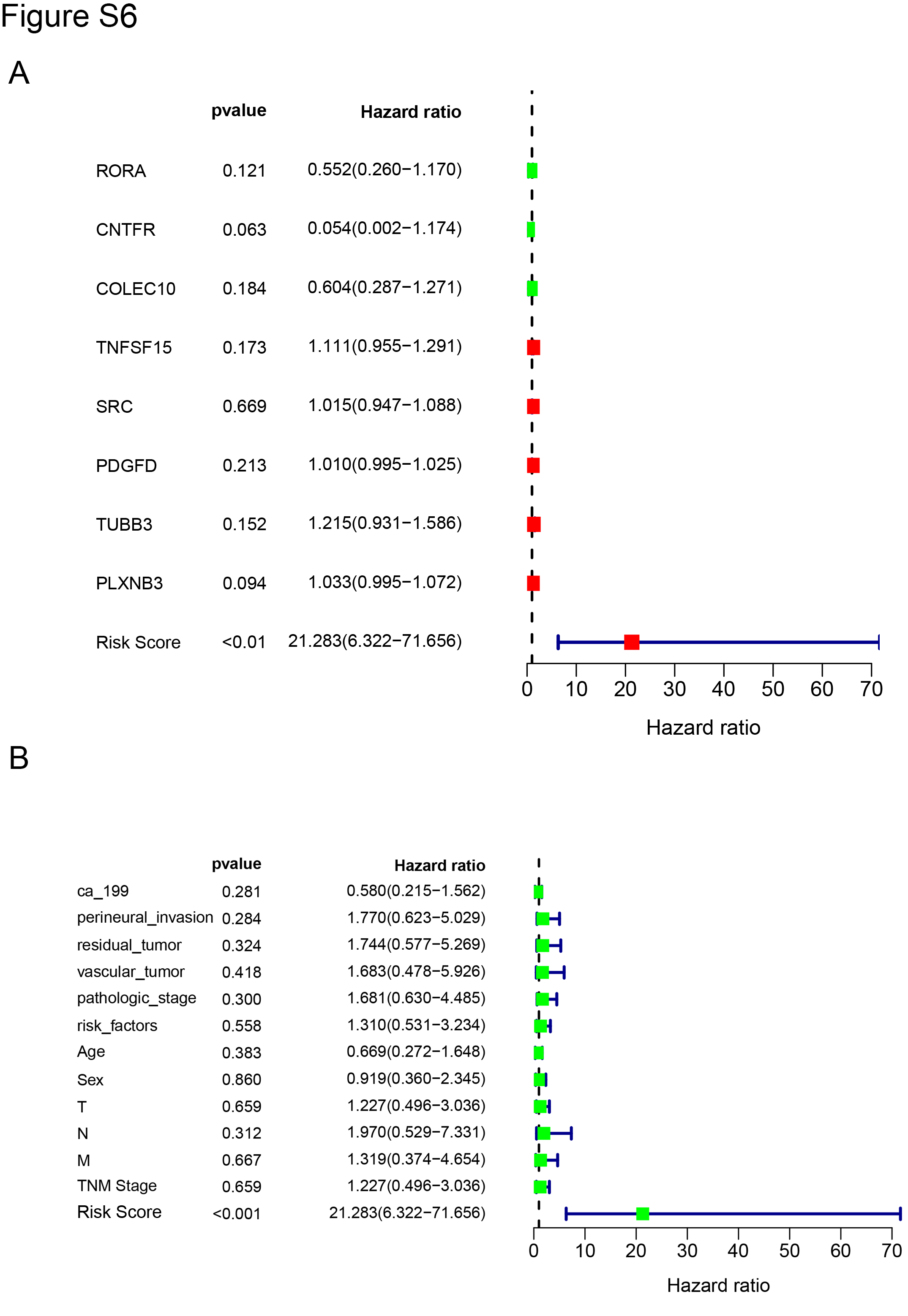

Supplement: Supplementary Figure 6 — Univariate survival analysis in the TCGA cohort. (A) Univariate survival analysis for single IRDEG and risk score. (B) Univariate survival analysis for clinical factor and risk score. [file Image_6.tif]

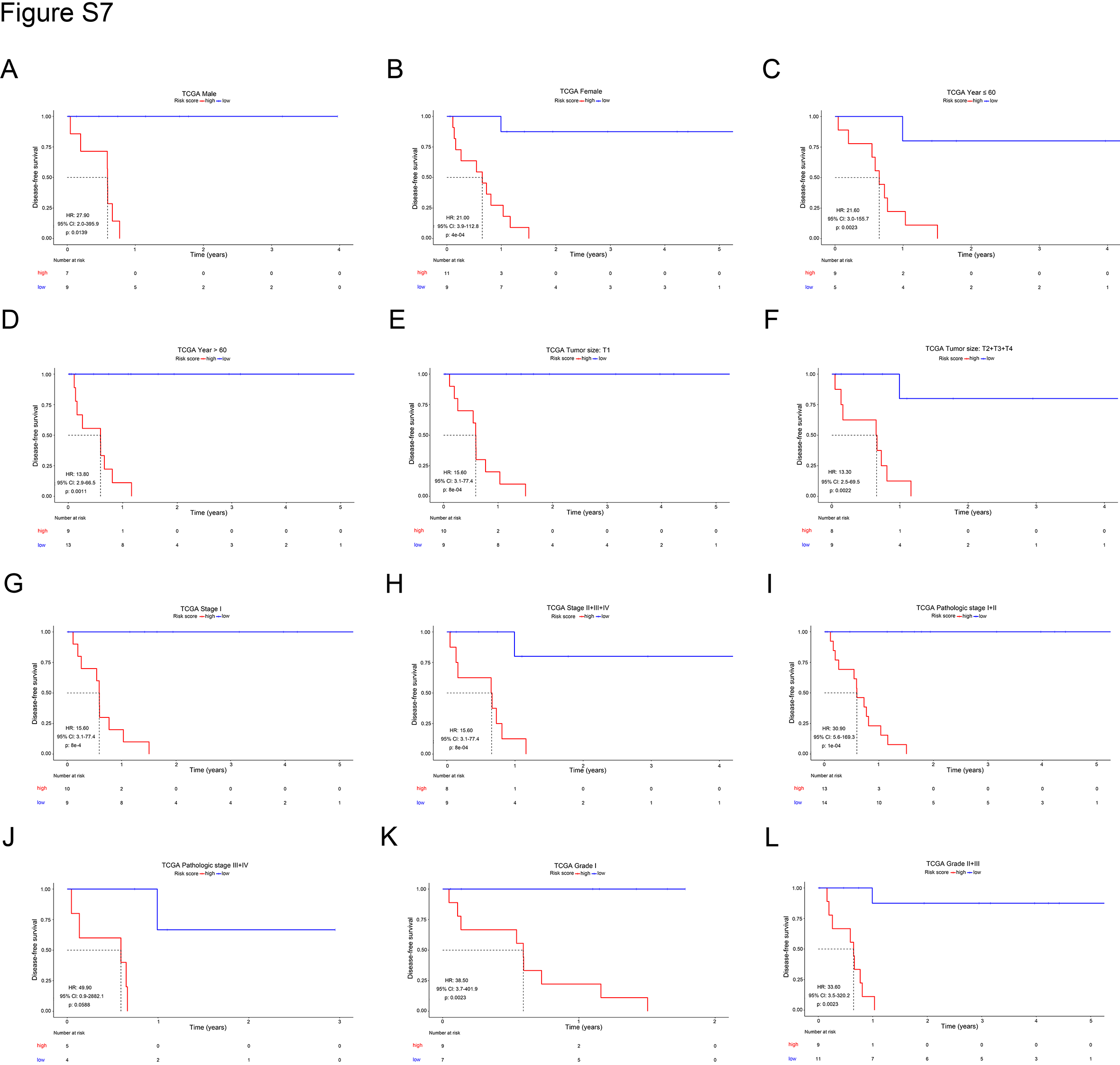

Supplement: Supplementary Figure 7 — Kaplan-Meier survival analyses of the TCGA cohort, according to the 8-IRDEGs-based classifier stratified by clinicopathological characteristics. (A, B) Gender, (C, D) Age, (E, F) Tumor size, (G, H) AJCC stage, (I, J) Pathologic stage, and (K, L) Grade. (P-values were calculated using the log-rank test. HR, hazard ratio). [file Image_7.tif]

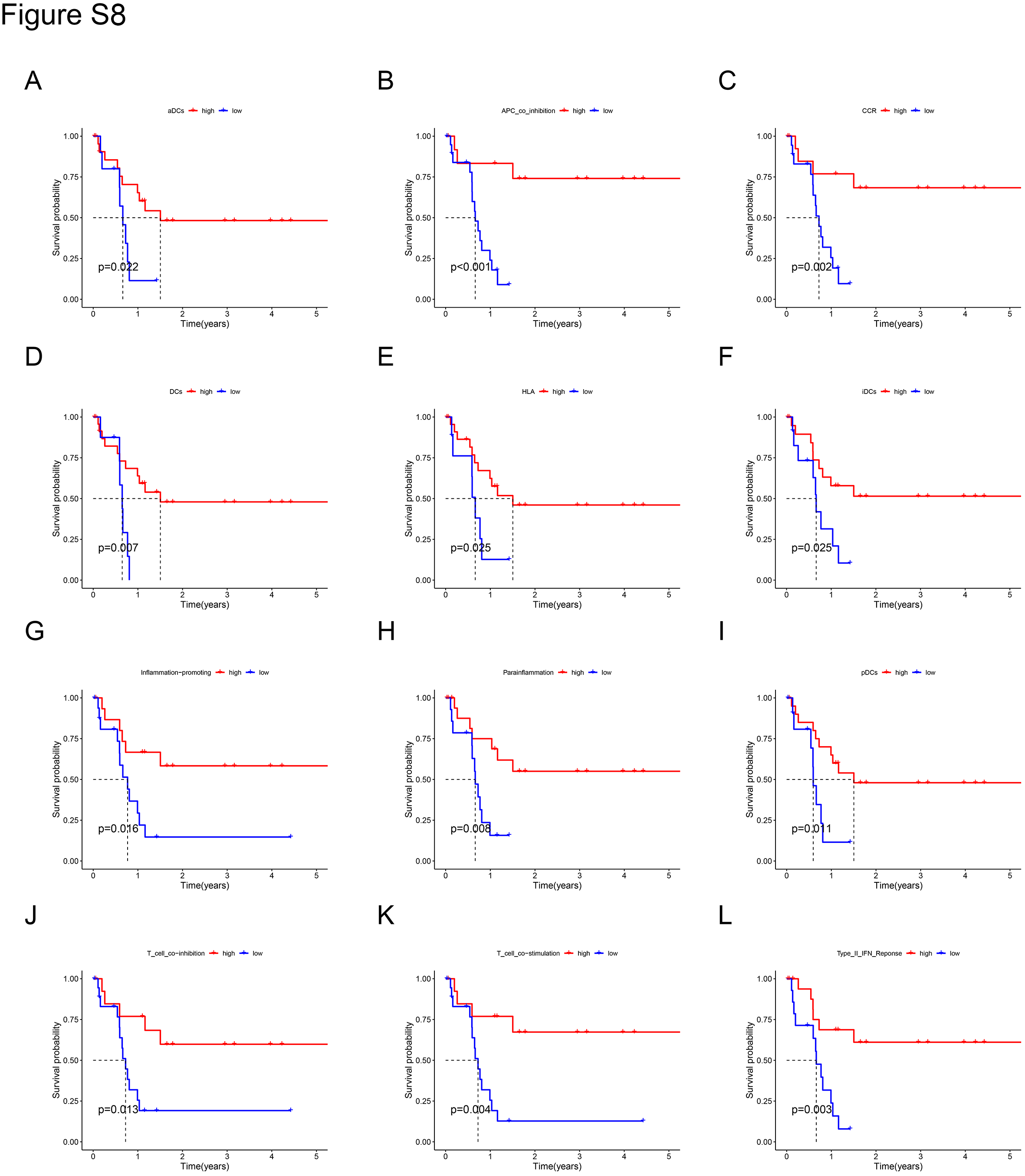

Supplement: Supplementary Figure 8 — The Kaplan-Meier survival analysis for innate immune functions. (A) The KM curve of the aDCs, (B) The KM curve of the APC-co-inhibition, (C) The KM curve of the CCR, (D) The KM curve of the DCs, (E) The KM curve of the HLA, (F) The KM curve of the iDCs, (G) The KM curve of the inflammation-promoting, (H) The KM curve of the Parainflammation. (I) The KM curve of the pDC, (J) The KM curve of the T-cell-co-inhibition, (K) The KM curve of the T-cell-co-stimulation, (L) The KM curve of the Type-II-IFN-Response. (P-values were calculated using the log-rank test. HR, hazard ratio). [file Image_8.tif]

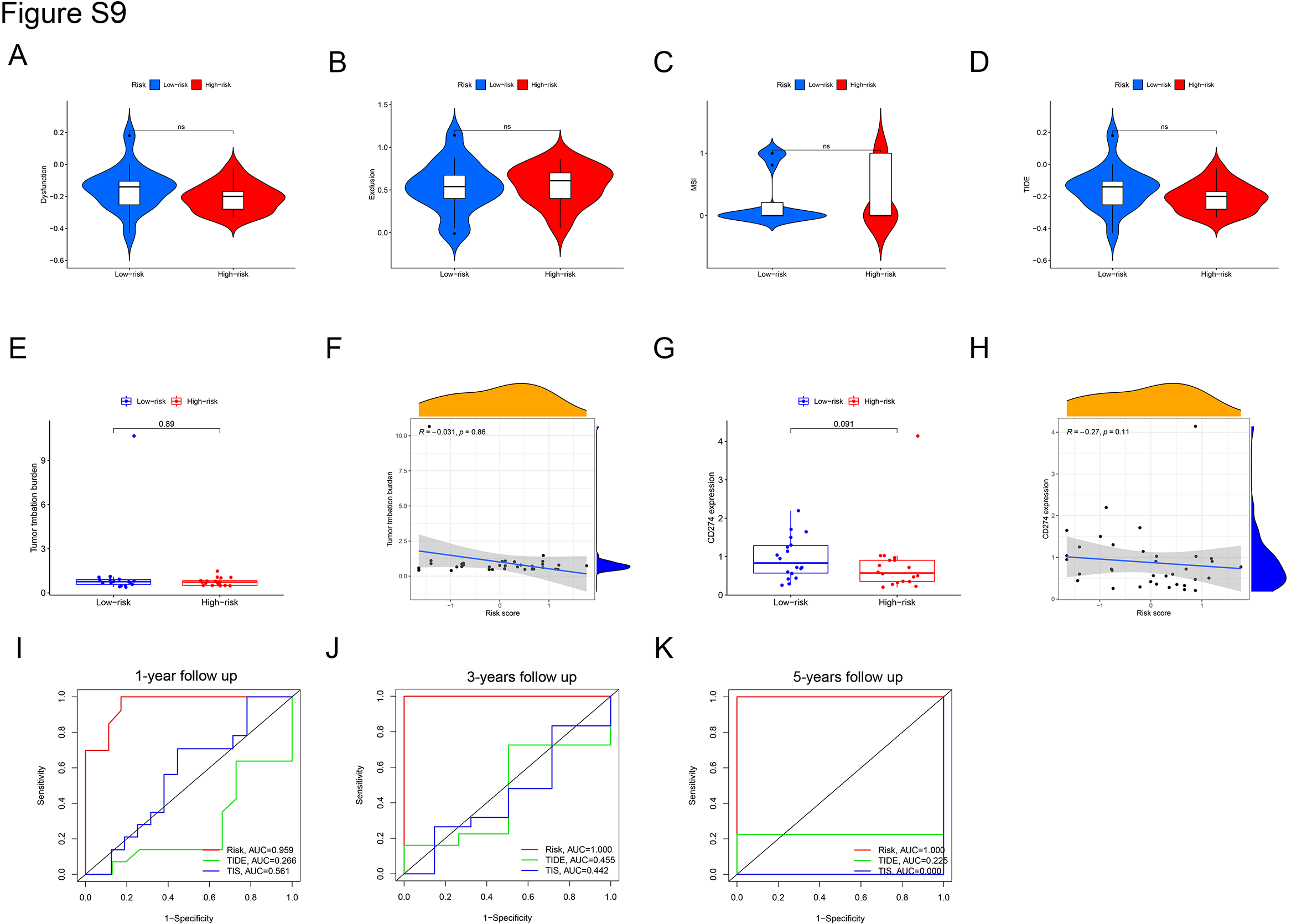

Supplement: Supplementary Figure 9 — The prognostic value of 8-IRDEGs signature in patients with anti-PD-L1 therapy. T cell dysfunction (A) and exclusion score (B), MSI (C), and TIDE (D) in different risk score subgroups. The score between the two subgroups were compared through the Wilcoxon test (ns: not significant, *p < 0.05, ** p < 0.01, *** p < 0.001). (E) TME in different risk score subgroups, (F) Correlations between the prognostic signature-derived risk score and TME. (G) PD-L1 in different risk score subgroups, (H) Correlations between the prognostic signature-derived risk score and PD-L1, (I–K) ROC analysis of 8-IRDEGs signature, TIS, and TIDE on DFS at 1- (I), 3- (J), and 5-years (K) follow-up. (person correlation analysis, AUC, area under ROC curve). [file Image_9.tif]

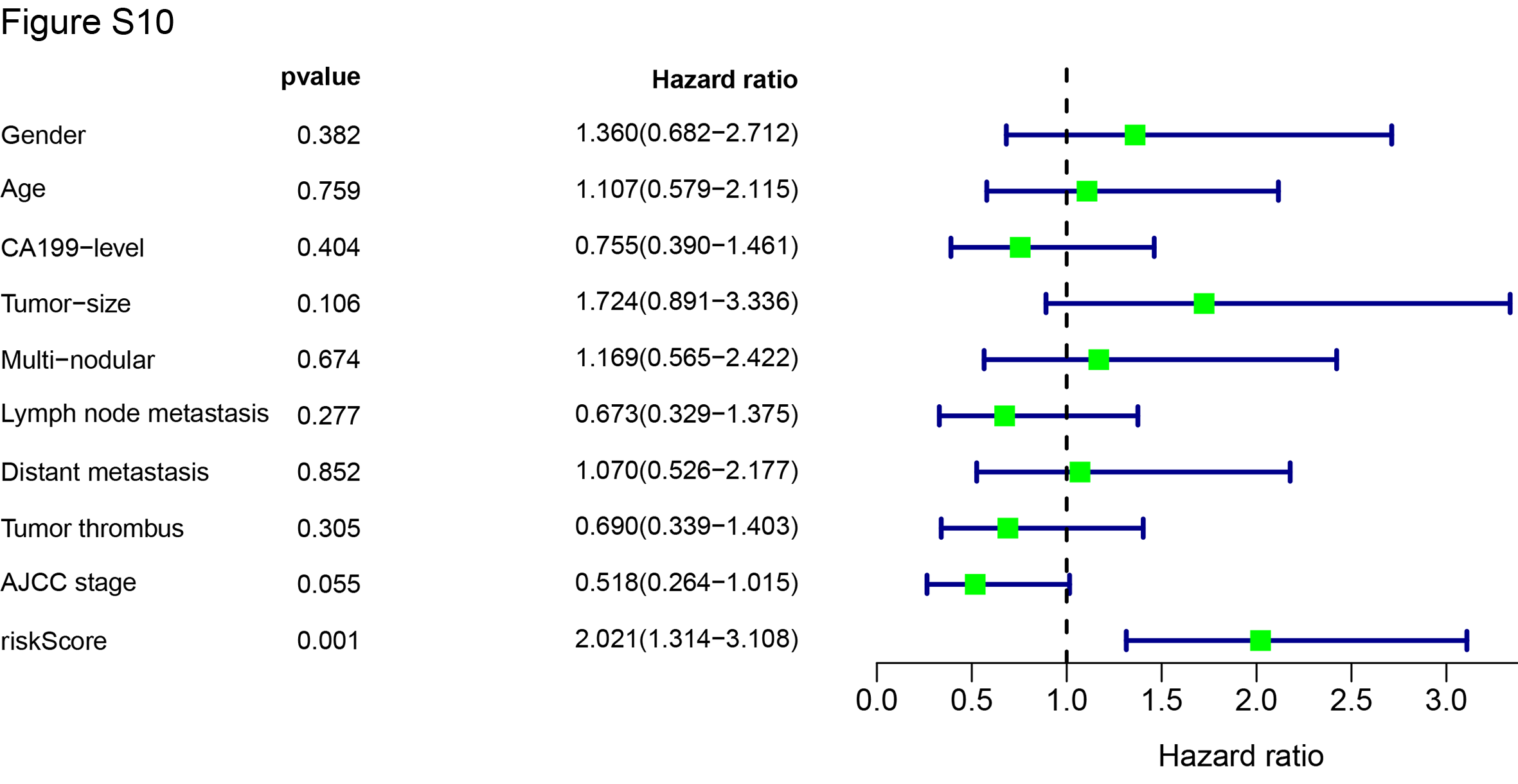

Supplement: Supplementary Figure 10 — Univariate survival analysis for the clinical factor and risk score in Renji Hospital. [file Image_10.tif]

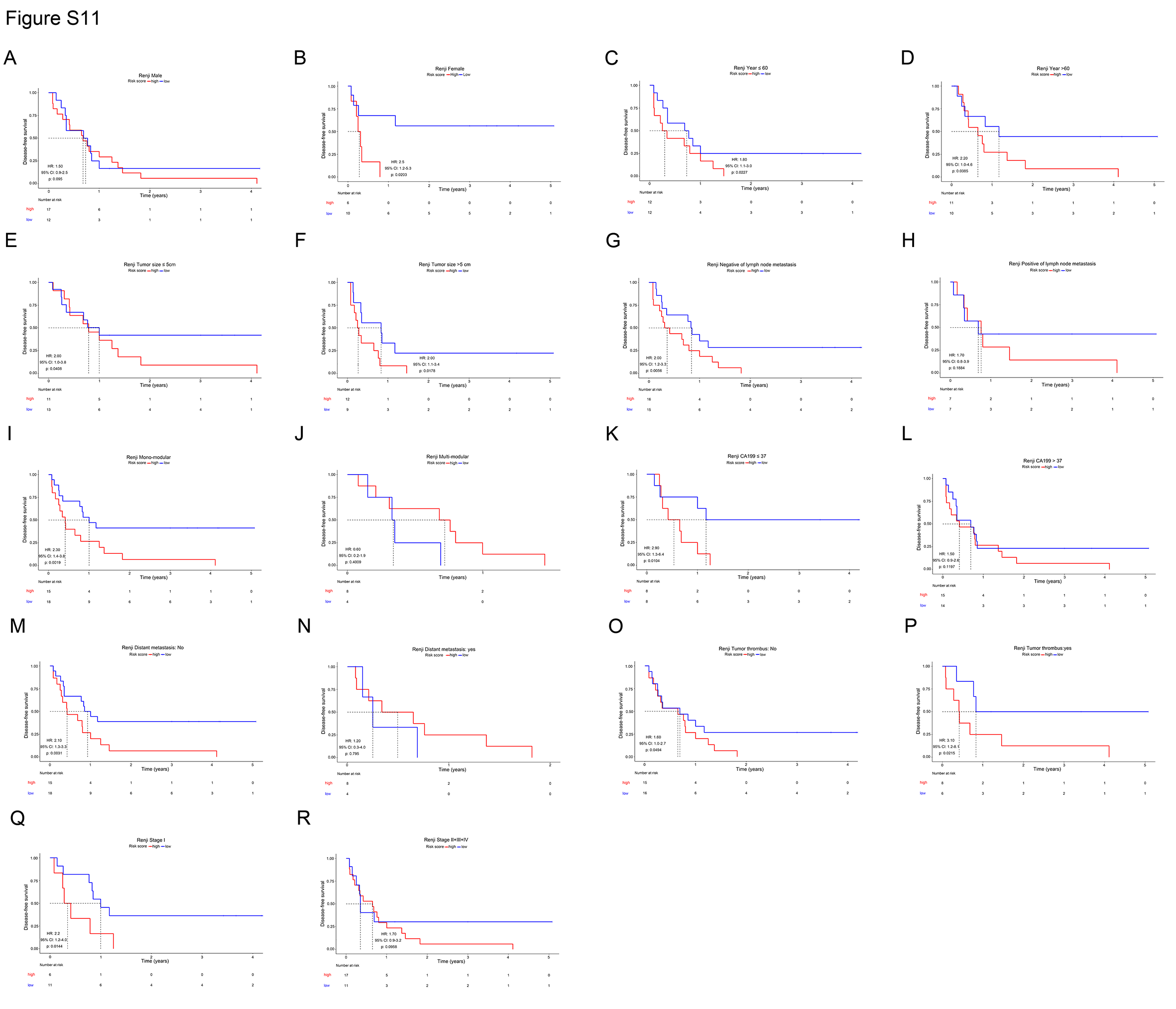

Supplement: Supplementary Figure 11 — Kaplan-Meier survival analyses of the Ren Ji cohort, according to the 8-IRDEGs -based classifier stratified by clinicopathological characteristics. (A, B) Gender, (C, D) Age, (E,F)Tumor size, (G,H) Lymph node metastasis, (I,J) Modular, (K, L) CA 19-9 level, (M,N) Distant metastasis,(O,P) Tumor thrombus,(Q,R) Stage. (P-values were calculated using the log-rank test. HR, hazard ratio). [file Image_11.tif]
