# Supplementary material for: An Immunity-Related Gene Model Predicts Prognosis in Cholangiocarcinoma
Source: Front Oncol. 2022 Jul 1;12:791867. doi: 10.3389/fonc.2022.791867 (PMC9283581; doi:10.3389/fonc.2022.791867)
Supplement: Supplementary file 12 [file Table_1.docx]

**Table S1. Clinical basic information of Ren Ji cohort.**

| **Number** | **Recurrence**  **(0, 1)** | **DFS**  **(Year)** | **Gender**  **(0, Female; 1, Male)** | **Age**  **(0, ≤60; 1, >60)** | **CA199 level**  **(0, ≤37; 1, >37)** | **Tumor size**  **(0, ≤5cm; 1, >5cm)** | **Multi-nodular**  **(0, ≤1; 1, >1)** | **Lymph node metastasis**  **(0, No; 1, Yes)** | **Distant metastasis**  **(0, No; 1, Yes)** | **Tumor thrombus**  **(0, No; 1, Yes)** | **AJCC stage**  **(0, I; 1, II+III+IV)** |
| --- | --- | --- | --- | --- | --- | --- | --- | --- | --- | --- | --- |
| RJ-01 | 1 | 1.37 | 0 | 1 | 1 | 0 | 0 | 0 | 1 | 0 | 1 |
| RJ-02 | 1 | 0.69 | 1 | 0 | 1 | 1 | 0 | 0 | 0 | 0 | 0 |
| RJ-03 | 1 | 0.77 | 0 | 0 | 1 | 1 | 0 | 0 | 0 | 0 | 0 |
| RJ-04 | 1 | 0.26 | 1 | 0 | 0 | 1 | 0 | 0 | 0 | 0 | 0 |
| RJ-05 | 1 | 0.42 | 1 | 1 | 1 | 1 | 1 | 1 | 1 | 0 | 1 |
| RJ-06 | 1 | 0.35 | 0 | 1 | 1 | 1 | 0 | 1 | 0 | 1 | 1 |
| RJ-07 | 1 | 0.09 | 0 | 0 | 1 | 0 | 0 | 0 | 0 | 0 | 0 |
| RJ-08 | 1 | 1.17 | 1 | 1 | 1 | 0 | 0 | 0 | 1 | 0 | 1 |
| RJ-09 | 1 | 0.34 | 1 | 1 | 1 | 1 | 1 | 1 | 0 | 0 | 1 |
| RJ-10 | 0 | 0.08 | 0 | 0 | 0 | 0 | 0 | 0 | 0 | 1 | 0 |
| RJ-11 | 1 | 0.68 | 1 | 1 | 0 | 1 | 0 | 0 | 1 | 0 | 1 |
| RJ-12 | 1 | 0.35 | 0 | 1 | 1 | 1 | 0 | 0 | 0 | 0 | 0 |
| RJ-13 | 1 | 0.21 | 0 | 1 | 1 | 0 | 0 | 0 | 0 | 0 | 0 |
| RJ-14 | 1 | 0.83 | 1 | 0 | 1 | 0 | 0 | 0 | 0 | 1 | 0 |
| RJ-15 | 1 | 0.08 | 1 | 0 | 0 | 1 | 1 | 0 | 1 | 0 | 1 |
| RJ-16 | 1 | 0.85 | 1 | 1 | 1 | 1 | 0 | 0 | 0 | 1 | 0 |
| RJ-17 | 0 | 3.67 | 0 | 0 | 0 | 0 | 0 | 0 | 0 | 0 | 0 |
| RJ-18 | 1 | 0.25 | 1 | 0 | 1 | 1 | 0 | 1 | 0 | 1 | 1 |
| RJ-19 | 0 | 4.20 | 0 | 1 | 0 | 0 | 0 | 0 | 0 | 0 | 0 |
| RJ-20 | 1 | 0.76 | 1 | 0 | 1 | 0 | 1 | 1 | 1 | 0 | 1 |
| RJ-21 | 0 | 3.00 | 1 | 0 | 1 | 0 | 1 | 0 | 0 | 0 | 1 |
| RJ-23 | 1 | 0.08 | 0 | 0 | 1 | 0 | 0 | 1 | 0 | 0 | 1 |
| RJ-24 | 0 | 3.42 | 0 | 1 | 0 | 1 | 0 | 0 | 0 | 0 | 0 |
| RJ-25 | 1 | 0.08 | 1 | 1 | 1 | 1 | 0 | 1 | 0 | 0 | 1 |
| RJ-26 | 1 | 1.00 | 1 | 1 | 1 | 0 | 0 | 1 | 0 | 1 | 1 |
| RJ-27 | 1 | 0.14 | 1 | 1 | 1 | 0 | 0 | 0 | 1 | 0 | 1 |
| RJ-28 | 1 | 0.65 | 1 | 0 | 1 | 0 | 0 | 0 | 1 | 1 | 1 |
| RJ-30 | 0 | 1.20 | 0 | 0 | 0 | 1 | 0 | 0 | 0 | 0 | 0 |
| RJ-31 | 1 | 0.79 | 1 | 1 | 1 | 0 | 0 | 0 | 1 | 0 | 1 |
| RJ-32 | 1 | 0.80 | 0 | 1 | 0 | 0 | 1 | 0 | 0 | 0 | 1 |
| RJ-33 | 1 | 0.31 | 1 | 0 | 1 | 1 | 1 | 0 | 0 | 1 | 1 |
| RJ-34 | 1 | 0.28 | 1 | 0 | 0 | 0 | 1 | 0 | 0 | 0 | 1 |
| RJ-35 | 1 | 0.33 | 1 | 0 | 1 | 1 | 1 | 1 | 0 | 1 | 1 |
| RJ-36 | 1 | 1.46 | 1 | 0 | 0 | 0 | 0 | 0 | 0 | 0 | 0 |
| RJ-37 | 1 | 1.25 | 1 | 1 | 1 | 0 | 0 | 0 | 0 | 1 | 0 |
| RJ-38 | 1 | 0.25 | 0 | 0 | 1 | 0 | 0 | 1 | 0 | 0 | 1 |
| RJ-39 | 1 | 0.08 | 0 | 0 | 1 | 1 | 0 | 1 | 1 | 0 | 1 |
| RJ-40 | 1 | 0.15 | 1 | 1 | 0 | 0 | 0 | 1 | 0 | 1 | 1 |
| RJ-41 | 1 | 1.00 | 1 | 1 | 0 | 1 | 0 | 0 | 0 | 1 | 0 |
| RJ-42 | 0 | 4.17 | 1 | 1 | 0 | 0 | 0 | 1 | 0 | 1 | 1 |
| RJ-43 | 0 | 5.08 | 1 | 0 | 0 | 0 | 1 | 0 | 0 | 1 | 1 |
| RJ-44 | 1 | 0.17 | 1 | 1 | 1 | 1 | 1 | 1 | 0 | 0 | 1 |
| RJ-45 | 1 | 4.11 | 0 | 0 | 1 | 1 | 1 | 0 | 1 | 0 | 1 |
| RJ-46 | 1 | 0.41 | 1 | 0 | 1 | 1 | 0 | 0 | 1 | 0 | 1 |
| RJ-47 | 1 | 1.82 | 1 | 0 | 0 | 0 | 0 | 0 | 0 | 0 | 0 |
