# Supplementary material for: An Immunity-Related Gene Model Predicts Prognosis in Cholangiocarcinoma
Source: Front Oncol. 2022 Jul 1;12:791867. doi: 10.3389/fonc.2022.791867 (PMC9283581; doi:10.3389/fonc.2022.791867)
Supplement: Supplementary file 13 [file Table_2.docx]

**Table S2. Primer sequences in the study.**

| **Gene symbol** | **Primer name** | **Primer sequences** |
| --- | --- | --- |
| **RORA** | homo-RORA-F | ACTCCTGTCCTCGTCAGAAGA |
|  | homo-RORA-R | CATCCCTACGGCAAGGCATTT |
| **CNTFR** | homo-CNTFR-F | CTGGGCTCTGACGTGACAC |
|  | homo-CNTFR-R | GTGGAAGCAGGCGTAGAGG |
| **COLEC10** | homo-COLEC10-F | ATAGCCGTCCTACCGCTGAA |
|  | homo-COLEC10-R | TGATCTCCCATATCACCCAGTTC |
| **TNFSF15** | homo-TNFSF15-F | TACTCCCAGGTCACATTCCGT |
|  | homo-TNFSF15-R | GGGTAGCTGTCTGTTACCTTGG |
| **SRC** | homo-SRC-F | GTGGACACTCAGGAGAAGAACG |
|  | homo-SRC-R | TGCTGCTTAATAATCTTGCCCTT |
| **PDGFD** | homo-PDGFD-F | TTGTACCGAAGAGATGAGACCA |
|  | homo-PDGFD-R | GCTGTATCCGTGTATTCTCCTGA |
| **TUBB3** | homo-TUBB3-F | GGCCAAGGGTCACTACACG |
|  | homo-TUBB3-R | GCAGTCGCAGTTTTCACACTC |
| **PLXNB3** | homo-PLXNB3 -F  homo-PLXNB3 -R | CGCTTCTCCGCACCTAATACC  CAGGGCTGTCGATTACAGGG |
