# Supplementary material for: An Immunity-Related Gene Model Predicts Prognosis in Cholangiocarcinoma
Source: Front Oncol. 2022 Jul 1;12:791867. doi: 10.3389/fonc.2022.791867 (PMC9283581; doi:10.3389/fonc.2022.791867)
Supplement: Supplementary file 14 [file Table_3.docx]

**Table S3. Clinical characteristics of patients according to the**

**8-IRDEGs signature classifier in the discovery cohort TCGA.**

| **Characteristics** | **Discovery Cohort**  **TCGA** | | **p-value** |
| --- | --- | --- | --- |
|  | **Low**  **(n=18)** | **High**  **(n=18)** |  |
| **Gender**  Female  Male | 9  9 | 11  7 | 0.502 |
| **Age (years)**  <60  ≥60 | 5  13 | 9  9 | 0.171 |
| **CA19-9 (ng/ml)^†^**  <37  ≥37 | 6  10 | 8  7 | 0.376 |
| **Tumor size (cm)**  <5  ≥5 | 9  9 | 10  8 | 0.738 |
| **Lymph node metastasis^†^**  No  Yes | 15  2 | 11  3 | 0.467 |
| **Distant metastasis^†^**  No  Yes | 14  3 | 14  2 | 0.680 |
| **Perineural invasion^†^**  No  Yes | 15  2 | 11  5 | 0.171 |
| **Residual tumor^†^**  R0  R1 | 14  1 | 14  4 | 0.215 |
| **Vascular tumor^†^**  No  Yes | 16  2 | 13  3 | 0.530 |
| **Pathologic stage**  I+II  III+IV | 14  4 | 13  5 | 0.700 |
| **TNM stage**  I  II+III+IV | 9  9 | 10  8 | 0.738 |

**^†^** The information was unavailable for some patients.
