# Supplementary material for: An Immunity-Related Gene Model Predicts Prognosis in Cholangiocarcinoma
Source: Front Oncol. 2022 Jul 1;12:791867. doi: 10.3389/fonc.2022.791867 (PMC9283581; doi:10.3389/fonc.2022.791867)
Supplement: Supplementary file 15 [file Table_4.docx]

**Table S4.** **Clinical characteristics of patients according to the**

**8-IRDEGs signature classifier in the discovery Ren Ji cohort.**

| **Characteristics** | **Discovery Cohort**  **Ren Ji hospital** | | **p-value** |
| --- | --- | --- | --- |
|  | **Low**  **(n=22)** | **High**  **(n=23)** |  |
| **Gender**  Female  Male | 10  12 | 6  17 | 0.175 |
| **Age (years)**  <60  ≥60 | 12  10 | 12  11 | 0.873 |
| **CA19-9 (ng/ml)**  <37  ≥37 | 8  14 | 8  15 | 0.912 |
| **Tumor size (cm)**  <5  ≥5 | 13  9 | 11  12 | 0.449 |
| **Multinodular**  No  Yes | 18  4 | 15  8 | 0.208 |
| **Lymph node metastasis**  No  Yes | 15  7 | 16  7 | 0.920 |
| **Distant metastasis**  No  Yes | 18  4 | 15  8 | 0.208 |
| **Tumor thrombus**  No  Yes | 16  6 | 15  8 | 0.586 |
| **AJCC stage**  I  II+III+IV | 11  11 | 6  17 | 0.098 |
